# Lifetime risk and multimorbidity of non-communicable diseases and disease-free life expectancy in the general population: A population-based cohort study

**DOI:** 10.1371/journal.pmed.1002741

**Published:** 2019-02-04

**Authors:** Silvan Licher, Alis Heshmatollah, Kimberly D. van der Willik, Bruno H. Ch. Stricker, Rikje Ruiter, Emmely W. de Roos, Lies Lahousse, Peter J. Koudstaal, Albert Hofman, Lana Fani, Guy G. O. Brusselle, Daniel Bos, Banafsheh Arshi, Maryam Kavousi, Maarten J. G. Leening, M. Kamran Ikram, M. Arfan Ikram

**Affiliations:** 1 Department of Epidemiology, Erasmus MC–University Medical Center Rotterdam, Rotterdam, the Netherlands; 2 Department of Neurology, Erasmus MC–University Medical Center Rotterdam, Rotterdam, the Netherlands; 3 Department of Psychosocial Research and Epidemiology, Netherlands Cancer Institute, Amsterdam, the Netherlands; 4 Department of Respiratory Medicine, Erasmus MC–University Medical Center Rotterdam, Rotterdam, the Netherlands; 5 Department of Bioanalysis, Faculty of Pharmaceutical Sciences, Ghent University, Ghent, Belgium; 6 Department of Epidemiology, Harvard T.H. Chan School of Public Health, Boston, Massachusetts, United States of America; 7 Department of Respiratory Medicine, Ghent University Hospital, Ghent, Belgium; 8 Department of Radiology and Nuclear Medicine, Erasmus MC–University Medical Center Rotterdam, Rotterdam, the Netherlands; 9 Department of Cardiology, Erasmus MC–University Medical Center Rotterdam, Rotterdam, the Netherlands; Stanford University, UNITED STATES

## Abstract

**Background:**

Non-communicable diseases (NCDs) are leading causes of premature disability and death worldwide. However, the lifetime risk of developing any NCD is unknown, as are the effects of shared common risk factors on this risk.

**Methods and findings:**

Between July 6, 1989, and January 1, 2012, we followed participants from the prospective Rotterdam Study aged 45 years and older who were free from NCDs at baseline for incident stroke, heart disease, diabetes, chronic respiratory disease, cancer, and neurodegenerative disease. We quantified occurrence/co-occurrence and remaining lifetime risk of any NCD in a competing risk framework. We additionally studied the lifetime risk of any NCD, age at onset, and overall life expectancy for strata of 3 shared risk factors at baseline: smoking, hypertension, and overweight. During 75,354 person-years of follow-up from a total of 9,061 participants (mean age 63.9 years, 60.1% women), 814 participants were diagnosed with stroke, 1,571 with heart disease, 625 with diabetes, 1,004 with chronic respiratory disease, 1,538 with cancer, and 1,065 with neurodegenerative disease. NCDs tended to co-occur substantially, with 1,563 participants (33.7% of those who developed any NCD) diagnosed with multiple diseases during follow-up. The lifetime risk of any NCD from the age of 45 years onwards was 94.0% (95% CI 92.9%–95.1%) for men and 92.8% (95% CI 91.8%–93.8%) for women. These risks remained high (>90.0%) even for those without the 3 risk factors of smoking, hypertension, and overweight. Absence of smoking, hypertension, and overweight was associated with a 9.0-year delay (95% CI 6.3–11.6) in the age at onset of any NCD. Furthermore, the overall life expectancy for participants without these risk factors was 6.0 years (95% CI 5.2–6.8) longer than for those with all 3 risk factors. Participants aged 45 years and older without the 3 risk factors of smoking, hypertension, and overweight at baseline spent 21.6% of their remaining lifetime with 1 or more NCDs, compared to 31.8% of their remaining life for participants with all of these risk factors at baseline. This difference corresponds to a 2-year compression of morbidity of NCDs. Limitations of this study include potential residual confounding, unmeasured changes in risk factor profiles during follow-up, and potentially limited generalisability to different healthcare settings and populations not of European descent.

**Conclusions:**

Our study suggests that in this western European community, 9 out of 10 individuals aged 45 years and older develop an NCD during their remaining lifetime. Among those individuals who develop an NCD, at least a third are subsequently diagnosed with multiple NCDs. Absence of 3 common shared risk factors is associated with compression of morbidity of NCDs. These findings underscore the importance of avoidance of these common shared risk factors to reduce the premature morbidity and mortality attributable to NCDs.

## Abstract 2

M. Arfan Ikram & colleagues assess the burden and preventability of co-occurring non-communicable diseases in the population-based Rotterdam Study.

## Author summary

**Why was this study done?:**

- The burden and preventive potential of disease is typically estimated for each non-communicable disease (NCD) separately, yet NCDs often co-occur, which hampers reliable quantification of their overall burden as well as the potential to prevent NCDs jointly in the general population.
- Smoking, hypertension, and overweight are 3 key risk factors that are shared by all NCDs, but their effects on the lifetime risk of developing any NCD, age at onset, and overall life expectancy with and without NCDs are uncertain.

**What did the researchers do and find?:**

- Between 1989 and 2012, we continuously followed 9,061 community-dwelling individuals in the Dutch population-based Rotterdam Study for occurrence of NCDs (stroke, heart disease, diabetes, chronic respiratory disease, cancer, and neurodegenerative disease).
- Nine out of 10 community-dwelling individuals aged 45 years and older will develop any NCD during their lifetime, with a third of them developing multiple NCDs during follow-up.
- Individuals without the 3 risk factors of smoking, hypertension, and overweight develop their first NCD on average 9 years later than those with all 3 risk factors.
- Absence of smoking, hypertension, and overweight is associated with a longer overall life expectancy of about 6 years, and a 2-year compression in lifetime spent with NCDs.

**What do these findings mean?:**

- In this western European community, we showed that the burden of NCDs in the general population is extensive and their multimorbidity is common.
- Absence of smoking, hypertension, and overweight is associated with a longer overall life expectancy, and most of this increase is due to an extension of disease-free life expectancy. This means that individuals without these risk factors not only live longer than individuals with these risk factors, but also spend less of their lifetime after the onset of symptomatic disease (which is referred to as compression of morbidity).
- These findings underscore the potential to substantially reduce premature NCD morbidity and mortality in the general population through prevention of smoking, hypertension, and overweight.

## Introduction

Non-communicable diseases (NCDs), including stroke, heart disease, diabetes, chronic respiratory disease, cancer, and neurodegenerative disease, are the most frequent causes of prolonged disability and premature death worldwide [1–3]. Major changes in lifestyle and medicine over the past decades have led to significant reductions in premature mortality from NCDs such as heart disease and cancer, especially in high-income countries [4,5], shifting the burden of disease in these countries from premature mortality to prolonged disability. In low- and middle-income countries, however, rates of premature mortality caused by NCDs are rapidly increasing, leading to severe socio-economic burdens in these societies [6].

The risks for most NCDs are highly modifiable, with the potential to halve lifetime risks through prevention of risk factor occurrence [7–10]. Avoidance of risk factors is referred to as primordial prevention, which is pivotal in reducing the growing burden of NCDs [11]. Primordial preventive efforts aim to eradicate risk factors before they occur, such as by maintaining a healthy weight in order to prevent overweight. In contrast, primary prevention is the reduction of risk factors that already exist, such as by efforts to lose weight in obese individuals. Population-based data on the multimorbidity of NCDs are needed to help in understanding the impact of risk factors on the lifetime risk and age at onset of NCDs. Three common shared risk factors—namely smoking, hypertension, and overweight—underlie most years spent with disability and the subsequent deaths caused by NCDs [12–14]. NCDs often co-occur, but few longitudinal data are available to inform us about patterns of multimorbidity in the general population [15,16]. Most studies that have assessed the burden of NCDs in the population have investigated each NCD separately [3,17,18], but the burden of multimorbidity—the coexistence of 2 or more chronic diseases—is now considered a global healthcare priority [16]. In view of continuing increases in life expectancies and improvements in healthcare systems worldwide, an increasing number of individuals are growing into old age, many of whom will survive their first NCD and become at risk for multimorbid age-related NCDs [19]. Thus, longitudinal data across the lifespan are required to better understand the clustering of NCDs. This understanding could help in shaping policy, public education, identification of individuals at increased risk of multimorbidity, and developing joint interventions to prevent multiple NCDs simultaneously. Mitigating shared risk factor burden is not only a cost-effective preventive strategy to curb the rapidly growing burden of NCDs [6], it is also the single most feasible way to meet one of the key Sustainable Development Goals: to reduce premature deaths from NCDs globally by a third by 2030 [20]. Long-term data on the occurrence of NCDs are useful to inform societies about the burden and multimorbidity of NCDs, and the potential to prevent multiple NCDs in the general population.

Lifetime risk and life expectancy are metrics that are relatively easy to interpret and can readily be used by relevant stakeholders. Importantly, these metrics can also capture the burden of NCDs and the potential for prevention in comparable detail to more complex metrics such as incidence rates and hazard ratios—thereby enabling policymakers, clinicians, and other stakeholders to expand current and future efforts aimed at preventing risk factors and reducing the growing burden of NCDs.

We used long-term data from a community-based, prospective cohort study to quantify the occurrence and multimorbidity of NCDs. We also calculated the lifetime risk of any NCD, accounting for NCD multimorbidity and the competing risk of death from other causes. Finally, we studied the association of shared risk factor burden with lifetime risk, age at onset of NCD, and life expectancy with and without NCDs.

## Methods

### Ethics statement

The Rotterdam Study has medical ethics committee approval per the Population Study Act: Rotterdam Study, executed by the Ministry of Health, Welfare and Sport of the Netherlands. Written informed consent was obtained from all participants. For the current study, the analysis plan was drafted in March 2018 (S1 Analysis Plan).

### Study population

This is a substudy from the Rotterdam Study, a prospective, population-based cohort study designed to assess the occurrence and determinants of age-related diseases in the general population [21]. In 1989 and 1990, all inhabitants aged 55 years and older from a well-defined suburb in the city of Rotterdam, the Netherlands, were invited to participate. This initial cohort comprised 7,983 participants. In 2000, 3,011 participants who had become 55 years of age, or had moved into the study district since the start of the study and were aged 55 years and older, were added to the cohort. In 2006, a further extension of the cohort was initiated in which 3,932 participants were included, aged 45 years and older. In total, the Rotterdam Study comprises 14,926 participants aged 45 years or over. The overall response rate across the 3 recruitment waves was 72% (14,926 out of 20,744 invitees).

To estimate lifetime risk of NCDs, we excluded 4,461 participants with a history of 1 or more NCDs at baseline (stroke *n* = 244, heart disease *n* = 915, diabetes *n* = 957, chronic respiratory disease *n* = 783, cancer *n* = 343, neurodegenerative disease *n* = 485, or a combination of these diseases *n* = 734). We further excluded participants who were incompletely screened at baseline for at least 1 of these diseases (*n* = 1,404), retaining 9,061 participants available for analyses.

### Risk factors

Smoking status was assessed during an in-depth interview, conducted by trained research assistants who visited the participants at home 2 weeks before their scheduled visit to attend the research centre. Smoking behaviour was categorised into never, former and current. At the research centre, blood pressure was measured at the right upper arm after at least 5 minutes’ rest in a seated position. Hypertension was defined as a resting sitting blood pressure exceeding 140/90 mm Hg (mean of 2 measurements) and/or the use of blood-pressure-lowering medication. Drugs categorised by the World Health Organization Anatomical Therapeutic Chemical (ATC) classification as antihypertensives (c02), diuretics (c03), beta blockers (c07), calcium channel blockers (c08), and RAAS-modifying agents (c09) were considered as blood-pressure-lowering medication. A body mass index ≥ 25 kg/m^2^ was considered as overweight. Marital status (living with or without partner) and educational attainment were also assessed during the same home interview to assess smoking status. Educational attainment was categorised as primary education (‘primary’), lower/intermediate general education or lower vocational education (‘lower’), intermediate vocational education or higher general education (‘further’), or higher vocational education or university (‘higher’).

### Ascertainment of NCDs

Baseline and follow-up ascertainment methods for stroke, heart disease (fatal or non-fatal coronary heart disease or heart failure), diabetes, chronic respiratory disease (chronic obstructive pulmonary disease or asthma), cancer (solid or haematological cancer), and neurodegenerative disease (dementia or parkinsonism) have previously been described in detail [9,22–26]. Disease-specific definitions, procedures, and data collection are summarised in S1 Methods. In brief, data on clinical outcomes are collected continuously through an automated follow-up system involving digital linkage of the study database to medical records maintained by general practitioners working in the research area. Trained research assistants affiliated with the study regularly check the medical records of each participant by hand for diagnoses of interest. Consultation notes, outpatient clinic reports, hospital discharge letters, electrocardiograms, pharmacy dispensing records, and imaging results are collected from general practitioner records and hospital records. Research physicians affiliated with the study independently review all data associated with events. Medical specialists also affiliated with the study review the potential cases, and adjudicate the final diagnosis in accordance with standardised diagnostic criteria. Further coverage of disease monitoring and subtyping is obtained through linkage to nationwide medical registries, the national cancer registry, and the Dutch pathology database. Information on vital status is obtained from the central registry of the municipality of the city of Rotterdam.

### Analysis

When individuals are followed for long time periods (e.g., from mid-life to occurrence of disease or death), preclusion of disease-specific outcomes of interest by death from other causes or by competing events may lead to overestimation of absolute risks in standard Kaplan–Meier analyses. To overcome the issue of such competing risks, we analysed the data taking into account the occurrence of competing events to compute remaining lifetime risks in left-truncated data, with age as the time scale [9,27,28]. Lifetime risk estimates reflect the competing-risk-adjusted cumulative incidences from that particular age onwards until the participant’s age at last follow-up. In this study, the maximum age was 106 years for men and 107 years for women.

First, we studied the occurrence and patterns of multimorbidity of NCDs during follow-up. We quantified the number of events for each NCD separately and visualised all observed combinations of NCD multimorbidity during follow-up with an intersection diagram [29].

Second, we calculated the combined cumulative incidences of these diseases from the age of 45 years to the participant’s age at last follow-up. The combined cumulative incidence equals the remaining lifetime risk of developing any NCD from the age of 45 years onwards. For these analyses, follow-up started at study entry (with the age of 45 years as minimum) and ended at the first date of diagnosis of any of the NCDs. This meant that we considered only the first occurring event of the 6 potential outcomes in this analysis in order to calculate the overall risk of developing any NCD, because when studying different risks of first manifestations of NCDs, the occurrence of 1 manifestation precludes consideration of any subsequent NCD event. For instance, participants who first experienced heart disease during follow-up were no longer considered as being at risk for stroke or any other NCD. This combined cumulative incidence of any NCD can be divided for each of the 6 diseases separately, which then represents the cumulative incidence of that specific disease occurring as the first manifestation of the 6 potential outcomes.

As a complementary analysis, we also calculated the disease-specific cumulative incidence, in which we considered only the disease of interest as the outcome, while disregarding the occurrence of the 5 other diseases. Thus, participants remained at risk of the 6 diseases irrespective of the occurrence of a first event. This meant that, for example, participants with heart disease during follow-up remained at risk for stroke in the analysis for stroke.

Third, we repeated these analyses stratified on the 3 risk factors at baseline, i.e., current smoking, hypertension, and overweight, to study whether these risk factors were related to overall lifetime risk. We also evaluated the effects of these risk factors on the age at onset of the first NCD and determined whether the type of disease as first manifestation differed across risk factor profiles.

Finally, we studied the effects of risk factors at baseline on life expectancy of participants with and without NCDs across absence and presence of risk factors using multistate lifetables [30]. This demographic tool combines all the life experiences of participants in 3 different health states: free of NCD, living with an NCD, and death. Transitions between these states could be from free of NCD to NCD (incident NCD), NCD to death (mortality among participants with NCD), and free of NCD to death (non-NCD mortality among participants without NCD). We considered only the first event into a state, and backflows were not allowed. For instance, for participants who developed multiple NCDs during follow-up, only their first NCD event (‘incident NCD’) is considered in these analyses. We adjusted for age, sex, birth year, marital status, and educational level. Detailed methodology for these calculations has been previously described, and is summarised in S1 Methods [30].

For the analyses on multimorbidity, study follow-up ended at date of death, loss to follow-up, or January 1, 2012, whichever came first. For the analyses on the lifetime risk of any NCD and on overall life expectancy, follow-up ended at the date of any incident NCD diagnosis (or, for the complementary disease-specific analysis, the date of incident NCD of interest), death, loss to follow-up, or January 1, 2012, whichever came first. Participants were considered lost to follow-up if they moved out of the Netherlands, if their medical records could not be accessed, or if participants withdrew their informed consent during follow-up. We used imputation procedures to impute missing data (<2.0%). This study is reported according to the Strengthening the Reporting of Observational Studies in Epidemiology (STROBE) guidelines (S1 Checklist). All analyses were performed at the significance level of 0.05 (2-tailed) using SPSS Statistics version 24.0.0.1 (IBM, Armonk, NY) and R version 3.4.3.

## Results

Study population characteristics are presented in Table 1. Median age at baseline was 61.7 years (range 45–107 years), and 60.1% of the population were women. Study population characteristics stratified by study wave are presented in Table A in S1 Results. Compared to participants included in the first study wave, participants in the second and third wave were generally younger at the start of follow-up. They attained a higher educational level, and were more likely to smoke or be overweight at baseline. Participants in the third wave were less likely to have hypertension at baseline than participants in the 2 other recruitment waves. During 75,354 person-years of follow-up (99.3% of potential person-years observed), 6,617 events occurred among 9,061 participants: 814 participants were diagnosed with stroke, 1,571 with heart disease, 625 with diabetes, 1,004 with chronic respiratory disease, 1,538 with cancer, and 1,065 with neurodegenerative disease. In total, 2,941 participants died during follow-up, of whom 421 died free of these diseases.

**Table 1:** Baseline characteristics of the study population.

Characteristic	All participants (*n* = 9,061)	Men (*n* = 3,603)	Women (*n* = 5,458)
Age (years), median (IQR)	61.7 (57.5–70.0)	61.2 (57.1–68.1)	62.2 (57.7–71.2)
Marital status			
Living with partner	6,427 (70.9%)	3,073 (85.3%)	3,354 (61.5%)
Living without partner	2,633 (29.1%)	530 (14.7%)	2,103 (38.5%)
Educational level			
Primary	1,476 (16.3%)	412 (11.4%)	1,060 (19.4%)
Lower	3,672 (40.5%)	1,048 (29.0%)	2,632 (48.2%)
Further	2,461 (27.2%)	1,278 (35.4%)	1,188 (21.8%)
Higher	1,452 (16.0%)	865 (24.0%)	578 (10.6%)
Smoking status			
Never	3,104 (34.3%)	569 (15.8%)	2,537 (46.4%)
Former	3,762 (41.5%)	1,952 (54.2%)	1,812 (33.2%)
Current	2,195 (24.2%)	1,082 (30.0%)	1,109 (20.3%)
Systolic blood pressure (mm Hg)	139 ± 21	140 ± 20	138 ± 21
Diastolic blood pressure (mm Hg)	79 ± 11	80 ± 11	78 ± 11
Use of blood-pressure-lowering medication	1,881 (20.8%)	627 (17.4%)	1,254 (23.0%)
Hypertension	4,696 (51.8%)	1,844 (51.2%)	2,852 (52.3%)
Body mass index (kg/m^2^)	26.9 ± 4.1	26.7 ± 3.4	27.0 ± 4.4
Overweight	5,751 (63.5%)	2,324 (64.5%)	3,427 (62.8%)
Years to first NCD diagnosis, median (IQR)	7.4 (3.5–11.9)	6.7 (3.3–11.2)	8.0 (3.7–12.5)
Presence of risk factors of smoking, hypertension, and overweight at baseline			
None of the 3 risk factors	1,343 (14.8%)	475 (13.2%)	868 (15.9%)
1	3,428 (37.8%)	1,334 (37.0%)	2,094 (38.4%)
2	3,656 (40.3%)	1,463 (40.6%)	2,193 (40.2%)
All 3 risk factors	634 (7.0%)	331 (9.2%)	303 (5.6%)

Imputed data presented as frequency (percent) for categorical values and mean ± SD for continuous variables, unless indicated otherwise. Data at baseline were virtually complete (<2.0% missing).

IQR, interquartile range; NCD, non-communicable disease; SD, standard deviation.

### Disease multimorbidity

**Fig 1:**
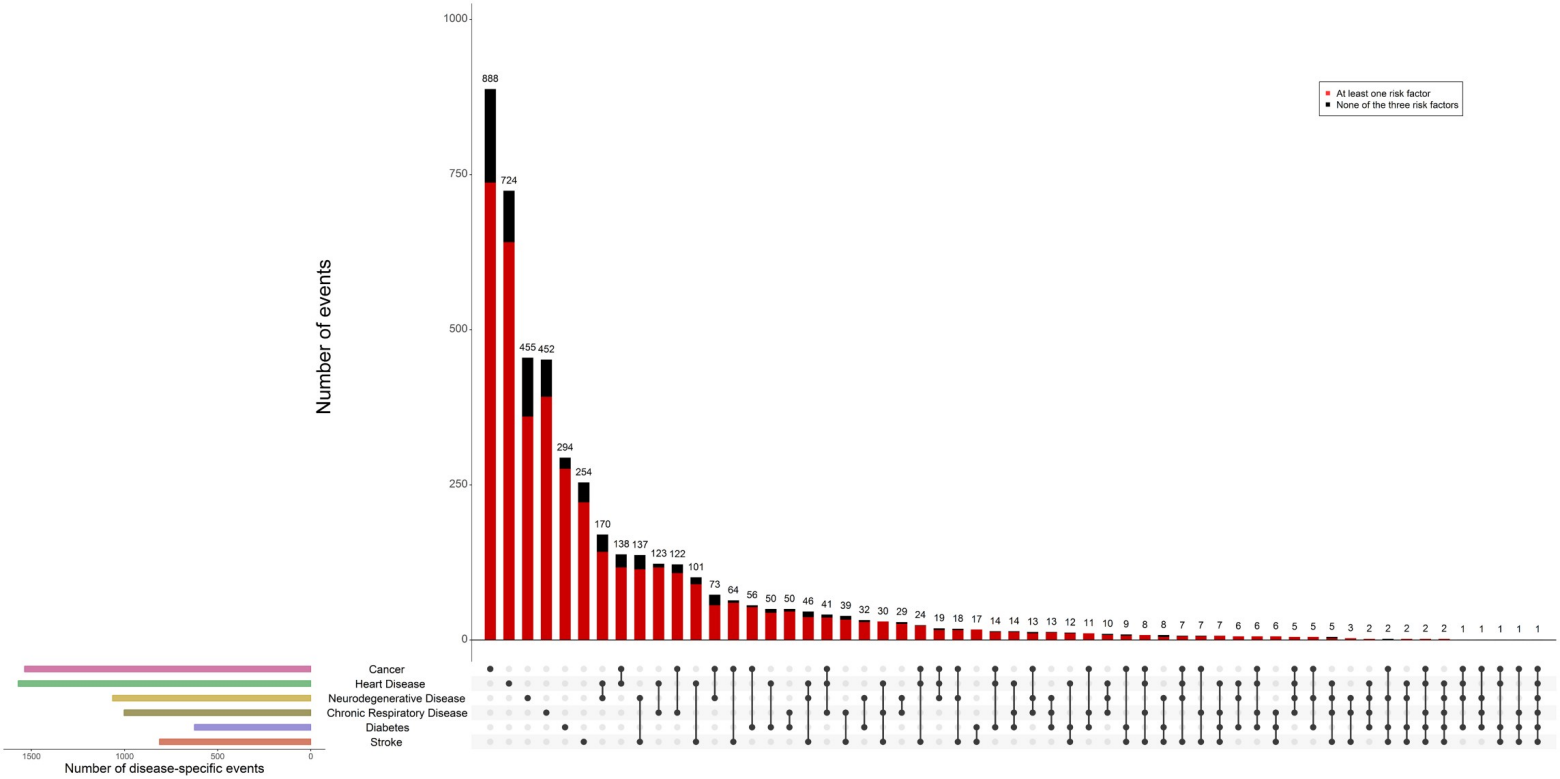
Intersection diagram depicting patterns of occurrence/co-occurrence of NCDs quantified as the number of events. Of all participants who developed an NCD during follow-up, a third (33.7%) were diagnosed with more than 1 of these diseases, including 1 individual who was diagnosed with all 6 diseases. The left panel displays bars for each disease separately that quantify the solitary number of events per disease, highlighting, for instance, that stroke occurred more frequently during follow-up than diabetes (814 versus 625 events, respectively). Yet stoke also showed more overlap with other NCDs compared to diabetes (254 solitary stroke events versus 294 solitary diabetes events). Events from participants who had at least 1 of the common shared risk factors (current smoking, hypertension, or overweight) at baseline are shown in red. NCD, non-communicable disease.

A third (33.7%) of all participants who developed an NCD (*n* = 4,633) were diagnosed with multiple NCDs during follow-up (Fig 1). The 5 most frequent clusters of disease were heart disease and neurodegenerative disease (*n* = 170; 3.7%), cancer and heart disease (*n* = 138; 3.0%), neurodegenerative disease and stroke (*n* = 137; 3.0%), heart disease and chronic respiratory disease (*n* = 123; 2.7%), and cancer and chronic respiratory disease (*n* = 122; 2.6%). Most participants who developed an NCD during follow-up were diagnosed with a single (*n* = 3,070; 66.3%) or 2 (*n* = 1,201; 25.9%) NCDs, whereas 1 individual was diagnosed with all 6 NCDs of interest. Among participants who developed a single NCD, cancer (*n* = 888; 28.9%) and heart disease (*n* = 724; 23.6%) were the most common, followed by neurodegenerative disease (*n* = 455; 14.8%), chronic respiratory disease (*n* = 452; 14.7%), diabetes (*n* = 294; 9.6%), and stroke (*n* = 254; 8.3%).

### Lifetime risk of any NCD

**Fig 2:**
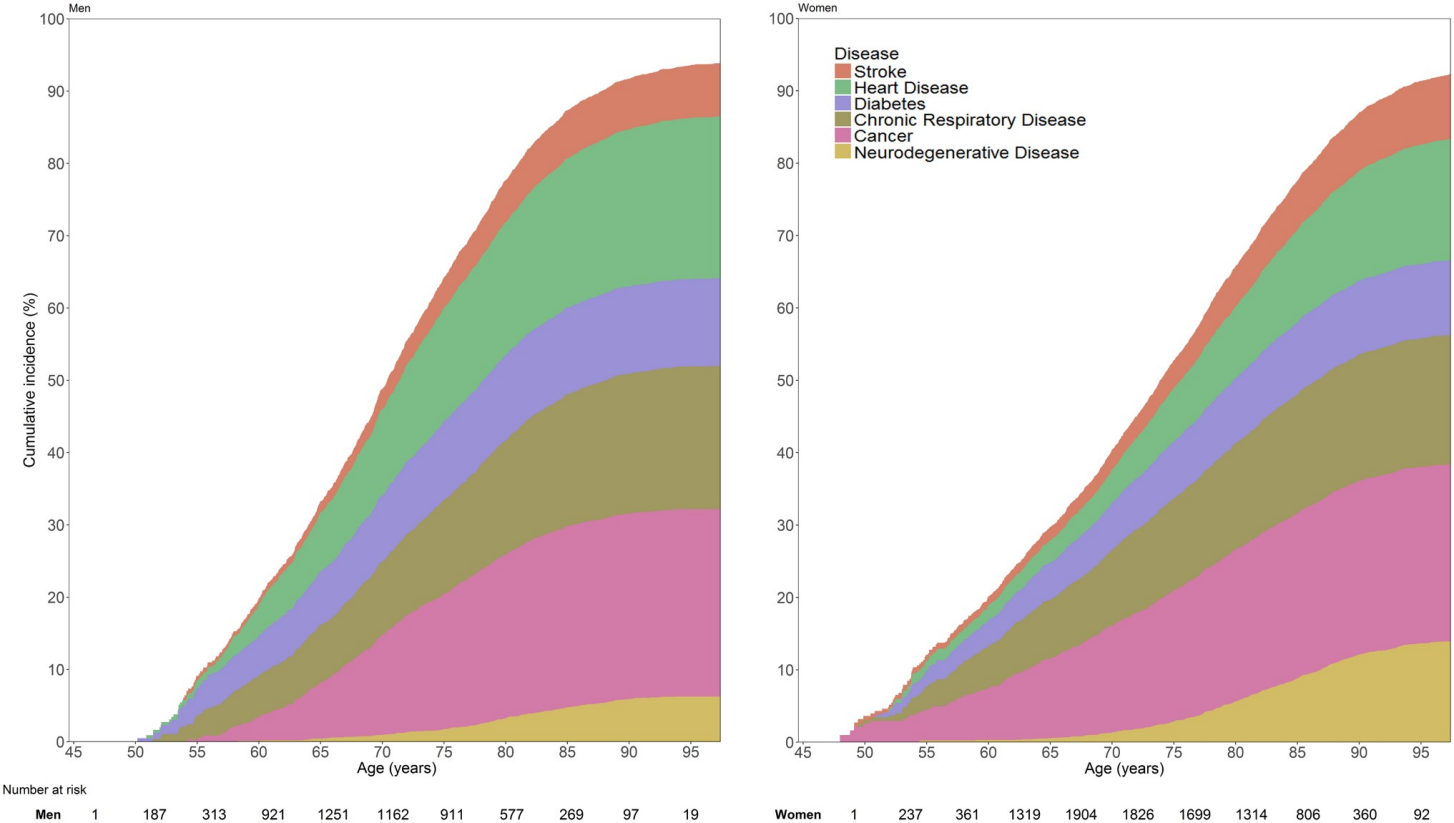
Lifetime risk of NCDs for 45-year-old men and women. In this analysis, follow-up ended at the time of first occurrence of an NCD. For instance, for participants who first experienced heart disease and subsequently developed neurodegenerative disease, only heart disease is considered here. NCD, non-communicable disease.

To calculate the lifetime risk of developing any NCD, we only considered the first NCD event during follow-up. In all, 1,173 participants were diagnosed with cancer as their first NCD, 1,035 with heart disease, 833 with chronic respiratory disease, 711 with neurodegenerative disease, 468 with stroke, and 413 with diabetes. In Fig 2, the combined cumulative incidence for developing any of these diseases from the age of 45 years onwards is presented for women and men separately. The risk of developing an NCD increased steeply with age, ranging from 33.3% for men and 29.8% for women between age 45 and age 65 years, up to 87.3% and 77.6%, respectively, until the age of 85 years. While men were at higher risk of developing an NCD at a younger age, the risk for women rapidly caught up with advancing age. Consequently, the overall lifetime risk of developing any of these diseases was only slightly higher for men compared to women, with a 94.0% (95% confidence interval (CI) 92.9%–95.1%) lifetime risk for a 45-year-old man and a 92.8% (95% CI 91.8%–93.8%) risk for a 45-year-old woman (*p-*value for sex difference < 0.001). This lifetime risk of developing any NCD remained stable across the included study waves (Table B in S1 Results). Dividing the combined cumulative incidence across the various NCDs showed that the difference in overall lifetime risk for these diseases between men and women was driven by a marked difference in risk for the type of first NCD. While cancer was the most common first NCD for both men (25.9%) and women (24.4%, *p-*value for sex difference = 0.22), the lifetime risk of heart disease as first NCD was significantly higher in men (22.5%) than women (17.0%, *p-*value for sex difference < 0.001). Conversely, the risk of neurodegenerative disease as first NCD was higher in women (14.1%) compared to men (6.3%, *p-*value for sex difference < 0.001). The complementary analysis, in which participants remained at risk for the specific NCD of interest irrespective of the occurrence of other NCDs, is presented in Table 2.

**Table 2:** Lifetime risks for each NCD separately, stratified by sex.

NCD	Percent (95% CI)		*p*-Value for-difference
	Men	Women	
Stroke	21.0 (18.4–23.5)	24.9 (22.5–27.2)	0.014
Heart disease	47.3 (44.6–50.0)	41.7 (39.4–44.1)	0.001
Diabetes	27.7 (24.2–31.3)	26.3 (23.5–29.1)	0.268
Chronic respiratory disease	37.5 (33.4–41.6)	27.4 (24.6–30.2)	<0.001
Cancer	48.4 (45.8–51.0)	37.3 (34.5–40.1)	<0.001
Neurodegenerative disease	23.7 (23.3–26.0)	35.9 (33.7–38.1)	<0.001

In these analyses, participants remained at risk for the specific NCD under study, irrespective of the occurrence of other NCDs, e.g., participants with an incident stroke or heart disease were still at risk of diabetes.

NCD, non-communicable disease.

### Impact of shared risk factors on lifetime risk and age at onset of NCDs

**Fig 3:**
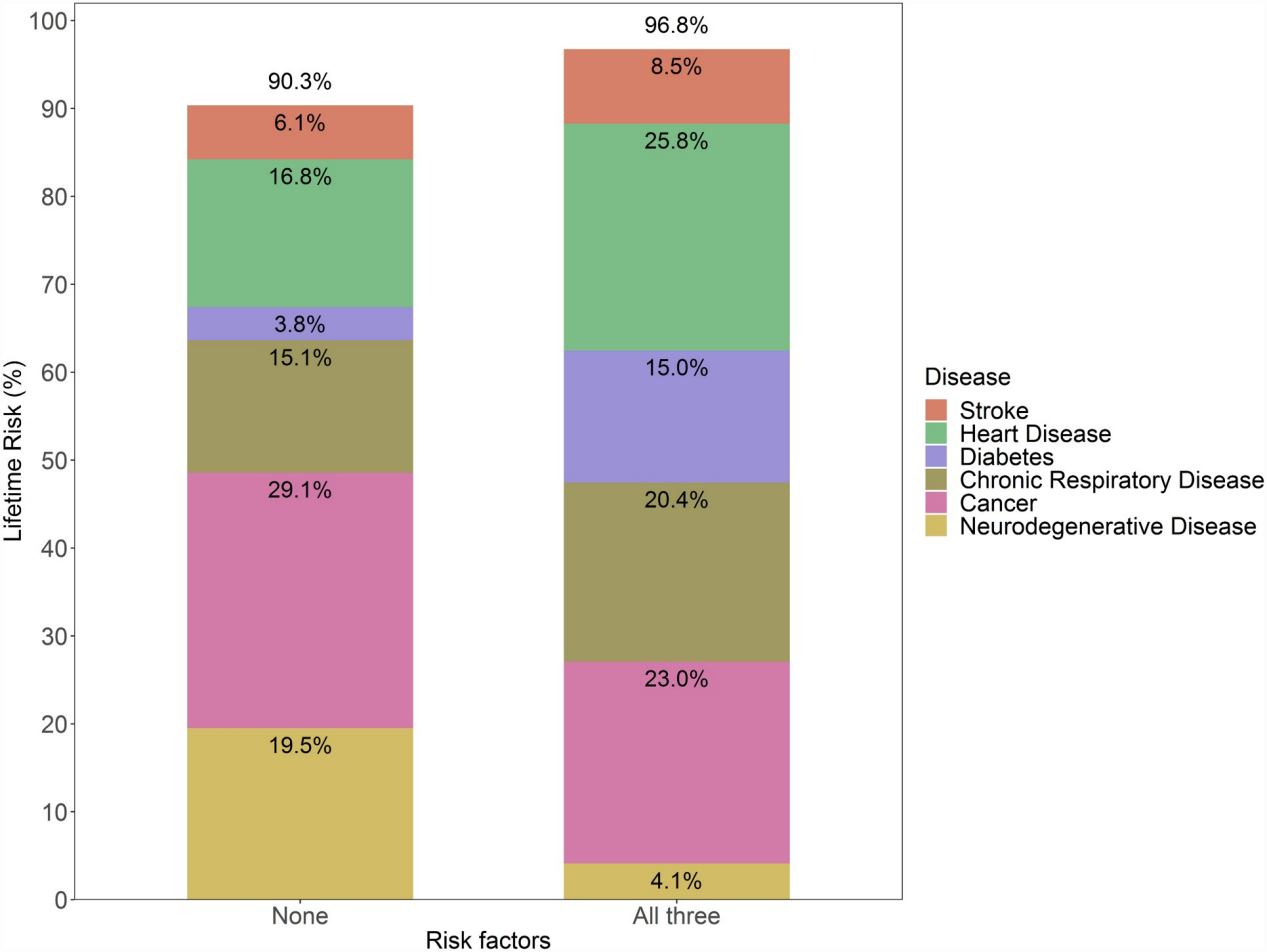
Lifetime risk of NCD stratified by risk factor burden. In this analysis, follow-up ended at the time of first occurrence of an NCD. For instance, for participants who first experienced heart disease and subsequently developed neurodegenerative disease, only heart disease is considered here. NCD, non-communicable disease.

**Fig 4:**
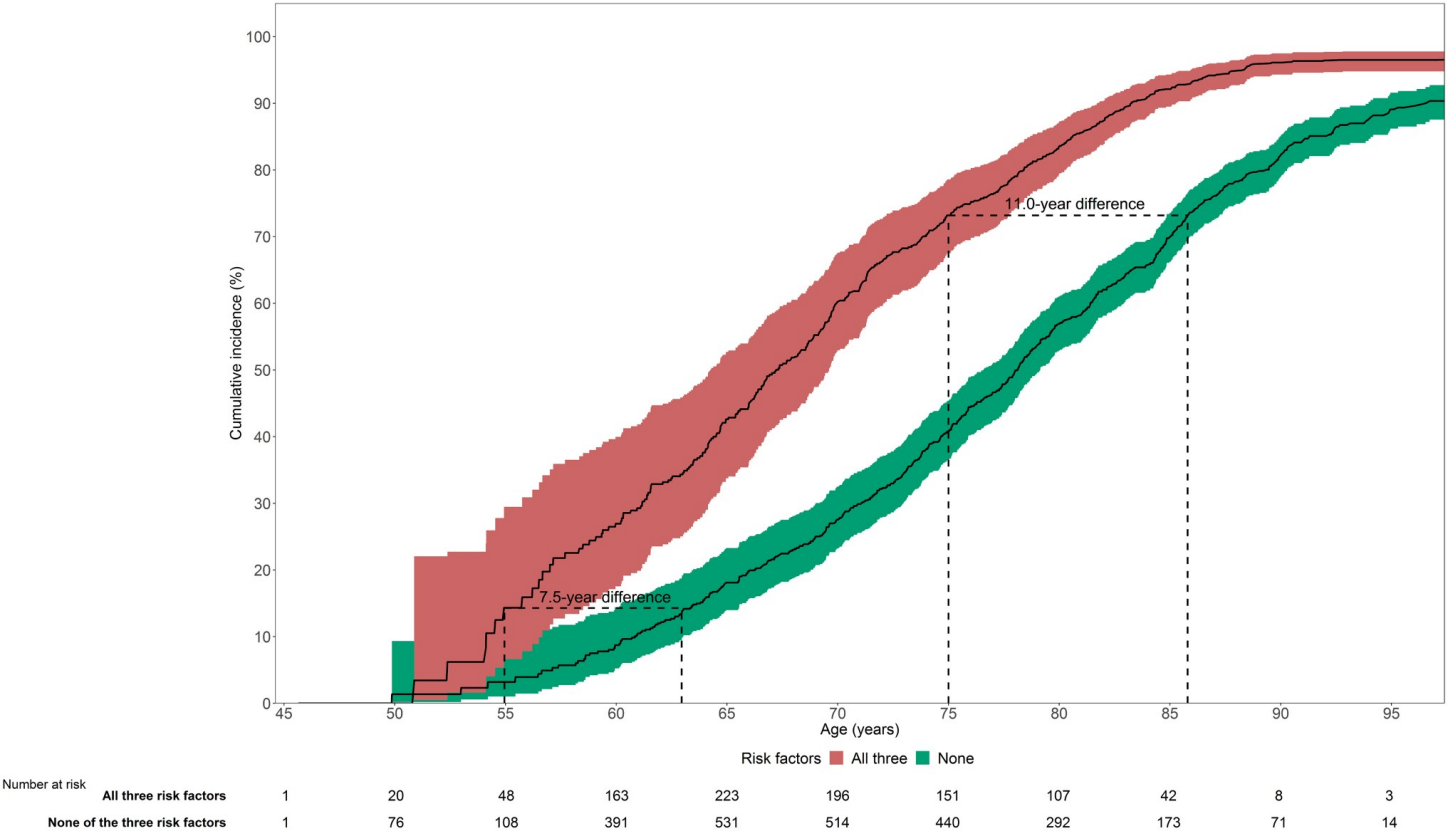
Cumulative incidence of NCD by risk factor burden. On average, a 9-year difference was observed in age at NCD onset between participants with all 3 risk factors (red) and those with no risk factors (green) at baseline. For example, at age 55 years, cumulative incidence was 14.3% for those with all 3 risk factors, whereas this cumulative incidence was not reached until age 62.5 years (i.e., 7.5 years later) for those without the risk factors. Similarly, at age 75 years, cumulative incidence was 73.2% for those with all 3 risk factors, whereas this cumulative incidence was not reached until age 86 years (i.e., 11 years later) for those without the risk factors. NCD, non-communicable disease.

When stratifying by risk factors, the overall lifetime risk of developing an NCD only slightly increased from 90.3% (95% CI 87.8%–92.9%) for those without the risk factors to 96.8% (95% CI 95.3%–98.2%) for participants with all risk factors (Fig 3). However, a large difference in the age at onset was observed (Fig 4): compared to participants who smoked, were hypertensive, and were overweight at baseline, participants without these risk factors were on average 9.0 years older (95% CI 6.3–12.6) when they were first diagnosed with an NCD. Similar trends were seen across the entire age range, for example, at age 55 years, cumulative incidence was 14.3% for those with all 3 risk factors, whereas this cumulative incidence was not reached until age 62.5 years (i.e., 7.5 years later) for those without the risk factors. Similarly, at age 75 years, cumulative incidence was 73.2% for those with all 3 risk factors, whereas this cumulative incidence was not reached until age 86 years (i.e., 11 years later) for those without the risk factors (Fig 4). These effects were accompanied by marked differences in the type of first NCD. For instance, participants without the 3 risk factors of smoking, hypertension, and overweight had a 16.8% lifetime risk of developing heart disease as a first manifestation, whereas this risk was 25.8% for those with all 3 risk factors. Participants without the risk factors remained at risk for neurodegenerative disease and cancer, while those with risk factors were particularly at risk for the development of heart disease, diabetes, and chronic respiratory disease. Compared to the joint effects of the 3 risk factors, differences in cumulative incidence and lifetime risk of developing any NCD became smaller when considering the effects of individual risk factors (Table C and Fig D in S1 Results). Similar patterns of risk factor effects were observed in disease-specific lifetime risk analyses (Table D and Fig E in S1 Results).

### Impact of shared risk factors on life expectancy with and without NCDs

**Fig 5:**
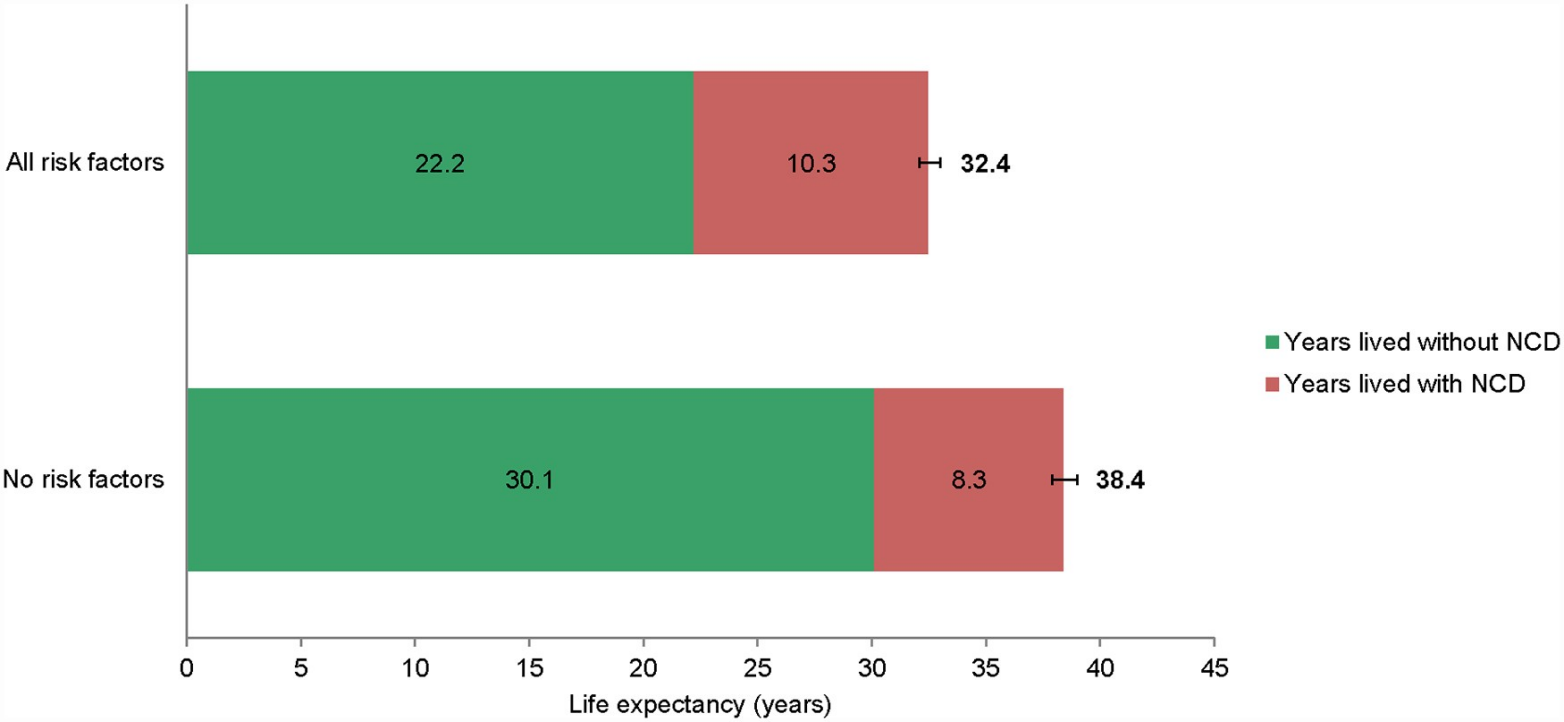
Remaining life expectancy at age 45 years with and without NCD stratified by risk factor burden. Participants aged 45 years and older without the 3 risk factors of smoking, hypertension, and overweight at baseline spent 21.6% (8.3 years divided by 38.4 years) of their remaining lifetime with at least 1 NCD, which was a substantially lower proportion compared to the 31.8% (10.3 years divided by 32.4 years) for those with all 3 of these risk factors at baseline. Error bars represent 95% confidence intervals. NCD, non-communicable disease.

Participants aged 45 years without the 3 shared risk factors smoking, hypertension, and overweight lived on average 6.0 years (95% CI 5.2–6.8) longer than those with all 3 of these risk factors (Fig 5). Moreover, while participants without these risk factors on average lived longer than those with these 3 risk factors, they also spent a smaller proportion of their life from the age of 45 years with at least 1 NCD. For instance, participants with the 3 risk factors of smoking, hypertension, and overweight spent on average almost a third (31.8%) of their remaining life expectancy from the age of 45 years with at least 1 NCD compared to approximately a fifth (21.6%) for those without these common shared risk factors. Similar trends in risk factor effects were observed when considering participants who had at least 1 or 2 of these risk factors (Figs E and F in S1 Results). Associations of individual risk factors with life expectancy were smaller compared to the joint effects of smoking, hypertension, and overweight (Table E and Fig G in S1 Results). When studying the individual association of smoking, hypertension, or overweight with life expectancy, participants who did not smoke had the longest life expectancy without an NCD (24.5 years [95% CI 24.3–24.7] compared to 20.0 years [95% CI 19.7–20.3] for participants who smoked).

## Discussion

In this population-based cohort study, a substudy of the ongoing prospective Rotterdam Study, we assessed the lifetime risks of developing co-occurring NCDs, and quantified their multimorbidity. We show that 9 out of 10 individuals develop an NCD from the age of 45 years onwards. Among those individuals, at least a third are subsequently diagnosed with multiple NCDs. Importantly, absence of 3 common shared NCD risk factors—namely smoking, hypertension, and overweight—is associated with a 9-year delay in the first diagnosis of any NCD compared to those with these 3 risk factors. Furthermore, absence of these risk factors is associated with an extended life expectancy of 6 years. These findings highlight the potential to lower the proportion of a lifetime spent with disability and the number of premature deaths caused by NCDs through prevention of shared risk factors among community-dwelling individuals.

Several cohort studies have assessed the disease-specific lifetime risks of various NCDs, including stroke [31,32], heart disease [10,28], diabetes [9], chronic respiratory disease [8,33], cancer [7] and neurodegenerative disease [32]. However, data were lacking on the combination of all these major NCDs within a single population and had not been used to calculate the overall lifetime risk of NCD among community-dwelling individuals. The extent of NCD multimorbidity and the effects of common shared risk factors on the onset of NCDs were largely unknown. Here, we show that as many as 9 out of 10 individuals aged 45 years and older will develop 1 or more of these NCDs during their remaining lifespan. In fact, according to our analyses that did not take multimorbidity into account, each of the 6 NCDs assessed in this study posed a high risk for community-dwelling individuals. For instance, the disease-specific remaining lifetime risk ranged from 21% for stroke in men up to 48% for cancer in men from the age of 45 years onwards. These numbers highlight the frequent occurrence of these NCDs in this western European population and show that—in view of the chronically progressive nature of these diseases—they are likely to impose a substantial burden on societies similar to the one in our study.

Modelling studies have shown the contribution of risk factor management to reducing NCD-related years spent with disability and (premature) mortality [17,18]. These studies were either based on prevalence data or relied on several assumptions, including the use of prediction models to estimate disease occurrence at the country level, and the derivation of relative risks from systematic reviews to quantify the effects of exposures on disease risk. Consequently, such analyses are hampered by the competing risks of death and other NCDs, the substantial multimorbidity of NCDs, and interactions between underlying risk factors. Indeed, several risk factors are shared between NCDs: most of the risk factors are, for instance, not only implicated in heart disease, but are also strongly related to other NCDs such as stroke, as well as an increased risk of mortality. If the competing risks of death and precluding NCD events are not appropriately accounted for, the risk of developing any NCD will be overestimated. For example, in our study, considering the lifetime risk of each NCD separately, without taking into account the competing risk of multimorbidity, results in a summed lifetime risk of the NCDs together exceeding 100%. Additionally, risk factors can often aggravate each other’s detrimental effects. Therefore, the preventive effect of removing multiple risk factors is often larger than the sum of the effects of removing the individual risk factors. Indeed, in our study the absence of smoking, hypertension, and overweight was far more beneficial in lowering disease risk and extending life expectancy than could be expected based on the individual associations of smoking, hypertension, or overweight alone.

In this study, we were able to overcome the aforementioned challenges by using real-world data, which allowed us to study the association of shared risk factors with the lifetime risk of developing any NCD, and with overall life expectancy with and without NCDs. Absence of the 3 shared risk factors of smoking, hypertension, and overweight was associated with a substantial delay in the onset of any NCD, and was also associated with a longer life expectancy free from NCDs. Results from this study in a western European population also suggest that many individuals residing in comparable populations with similarly organised healthcare systems will be affected by an NCD at some point in their life, although at what age and which disease type will manifest first are strongly influenced by an individual’s underlying risk factor profile. We found substantial differences in first manifestation of NCD between men and women. Although men and women have a roughly similar lifetime risk of developing any NCD, men are more likely to develop NCDs at a younger age, and to develop heart disease, chronic respiratory disease, or diabetes as their first event. Women are more likely to develop their first NCD at an older age, and subsequently have a higher risk of stroke and neurodegenerative disease, as compared with men. These results extend prior findings on sex differences in lifetime risk and first manifestation of heart disease, stroke, and neurodegenerative disease [28,32].

Some limitations of this study must be acknowledged. First, findings from this community-based study of predominantly white individuals from western Europe have limited generalisability to capture the contemporary burden of NCDs in low- and middle-income countries. Nevertheless, given the expected increase in life expectancy in coming years in low- and middle-income countries, these results may be informative to help the societies of these countries prepare future resource allocation. Second, although the overall response rate in this study was high (72%), non-responders may have had higher than average risk factor burden and associated risk of NCDs, which may have led to some underestimation of results. Third, in the Dutch healthcare system, the entire population is entitled to primary care that is covered by their (obligatory) health insurance. In this primary care setting, a general practitioner provides primary prevention for NCDs, which may have affected the risk, age at onset, and type of NCD. Generalising the results of our study to healthcare systems that are organised differently, or to those that have limited availability of primary preventive healthcare, should therefore be done with caution. Fourth, risk factors were ascertained at baseline, which does not capture the possibility of individuals developing additional risk factors during follow-up or, conversely, transitioning from an adverse to a more optimal risk profile. Finally, individuals who do not smoke or have hypertension and are not overweight might also have other factors associated with a healthy lifestyle, such as a healthy diet and physical activity. Indeed, residual confounding may have influenced the results, which limits causal interpretation of findings from this observational study. Nonetheless, a major strength of this study is the long-term follow-up of multiple NCDs systematically assessed in a single contemporary population study. This allowed the quantification of NCD multimorbidity, the calculation of the lifetime risk of developing any NCD, and the calculation of life expectancy with and without NCD.

Over the past decades, smoking control and treatment of hypertension, along with improvements in treatment options, have led to a marked decline in premature deaths from heart disease despite clear trends in increased prevalence of overweight [34]. Our results indicated that the absence of smoking, hypertension, and overweight was associated with a 9-year delay in the age at onset of NCD, compared to individuals with these risk factors. This delay in the age at onset of NCD remained roughly similar across the entire age span, and subsequently led to marked differences in first manifestation of NCD. Individuals with the most adverse risk factor profiles had an approximately doubled risk of developing chronic respiratory disease, diabetes, or heart disease as first NCD. Conversely, healthy individuals with optimal risk factor levels were more likely to develop an NCD at older age, and as a consequence had a 5-fold increased risk of developing neurodegenerative disease as their first manifestation. Life expectancy analyses showed that individuals without the common shared risk factors at baseline lived longer without an NCD, yet lived for a shorter time after diagnosis of an NCD. Individuals without these 3 risk factors had a 6-year longer overall life expectancy than people with these risk factors, and they also spent much more of their remaining life expectancy from the age of 45 years free from NCD compared to individuals with these 3 risk factors. As such, individuals without the risk factors gained relatively more healthy life years than years of overall life expectancy, and as a consequence, the time between the onset of chronic illness or disability and the moment of death was compressed. This is referred to as compression of morbidity [35]. In summary, the findings of this study emphasize that efforts aimed at optimal prevention of common shared risk factor occurrence may benefit healthy aging at a population level.

### Conclusions

In this cohort study of a western European community, 9 out of 10 individuals aged 45 years and older develop an NCD during their remaining lifetime. Among those individuals, at least a third are subsequently diagnosed with multiple NCDs. Absence of the 3 shared risk factors of smoking, hypertension, and overweight is associated with a 9-year delay in the age at onset of any NCD, and a significantly prolonged life expectancy. These findings highlight the potential to reduce premature disability and death caused by NCDs through primordial prevention of smoking, hypertension, and overweight.

## Supporting information

S1 Analysis Plan The pre-specified statistical analysis plan. (PDF)

S1 Checklist STROBE reporting checklist for cohort studies. (DOCX)

S1 Methods Supplementary information on the methods used in this study. (DOCX)

S1 Results Additional analyses on lifetime risk of NCDs and life expectancy with and without NCD. (DOCX)

## Abbreviation

CI: confidence interval

NCD: non-communicable disease
