# Supplementary material for: Lifetime risk and multimorbidity of non-communicable diseases and disease-free life expectancy in the general population: A population-based cohort study
Source: PLoS Med. 2019 Feb 4;16(2):e1002741. doi: 10.1371/journal.pmed.1002741 (PMC6361416; doi:10.1371/journal.pmed.1002741)
Supplement: S1 Analysis Plan — (PDF) [file pmed.1002741.s001.pdf]

STATISTICAL ANALYSIS PLAN

Lifetime risk and multimorbidity of non-communicable diseases and disease-free life expectancy in the general population: A population-based cohort study

Date: March 2018

Version: 2.0

Background & Rationale

Non-communicable diseases (NCDs), including stroke, heart disease, diabetes, chronic respiratory disease, cancer and neurodegenerative disease, are the most frequent causes of prolonged disability and premature death worldwide. Most NCDs are highly amendable with the potential to halve lifetime risks through prevention of risk factor occurrence. The avoidance of risk factors is referred to as primordial prevention, which is pivotal in order to reduce the growing burden of NCDs. Population-based data on the co-occurrence of NCDs are lacking, but urgently required to properly study the impact of risk factor presence on the lifetime risk and age at onset of NCDs. Three common shared risk factors, namely smoking, hypertension, and overweight, underlie most of the years spent with disability, and the subsequent deaths caused by NCDs.

Mitigating shared risk factor burden is not only a cost-effective preventive strategy to curb the rapidly growing burden of NCDs, it is presumably also the single most feasible way to meet one of the key Sustainable Development Goals to globally reduce premature deaths from NCDs by a third by 2030. In order to timely attain this goal, long-term data on (co-)occurrence patterns of all NCDs are useful to inform societies about the risk, and the potential of postponing, or even preventing their onset collectively through primordial prevention of common shared risk factors.

Lifetime risk and life expectancy are comprehensible, yet accurate metrics to capture the burden of NCDs, and their preventive potential in great detail. This would subsequently prompt policymakers, clinicians, and other stakeholders to expand current and future efforts aimed at optimal prevention of risk factor occurrence to reduce the growing burden of NCDs.

In the present study, we will use data from the community-based, prospective cohort Rotterdam Study to calculate the lifetime risk of any NCD, while accounting for their co-occurrence, and competing risk of death from other causes. We will also study the association of shared risk factor burden with lifetime risk, age at onset, and life expectancy with and without NCDs.

Objectives

A. To study patterns of co-occurrence of NCDs in the general population.
B. To calculate the overall lifetime risk of developing any NCD.
C. To calculate disease-specific lifetime risks of the different manifestations of NCDs.
D. To study associations of three common shared risk factors, namely smoking, hypertension and overweight, with lifetime risk, age at onset, and life expectancy with and without NCDs.

Study design

This study will be embedded within the Rotterdam Study, a prospective population-based cohort designed to study the occurrence and determinants of age-related diseases in the general population. In 1990, all inhabitants aged 55 and older from a well-defined suburb in the city of Rotterdam, the Netherlands were invited to participate. This initial cohort compromised 7,983 participants. In 2000, 3,011 participants who had become 55 y of age or moved into the study district since the start of the study if aged 55 y and older, were added to the cohort. In 2006, a further extension of the cohort was initiated in which 3,932 participants were included, aged 45 y and older. In total, the Rotterdam Study comprises 14,926 participants aged 45 y or over. The overall response rate across the three recruitment waves was 72%.

Study population

The study population will consist of participants from the aforementioned Rotterdam Study, recruited across three the study waves: RS-I-1 (1990-1993, N=4,869), RS-II-1 (2000-2001, N=1,620) and RS-III-1 (2006-2008, N=2,572), who are free from NCDs at baseline and who have provided informed consent to access medical records at baseline and during follow-up.

Primary outcome

Incident NCD (stroke, heart disease, cancer, chronic respiratory disease, diabetes or neurodegenerative disease) or death due to other causes.

Statistical analysis

Baseline characteristics

Baseline characteristics will be descriptively summarized for all included participants, and stratified by sex. Group means will be used for group comparison using t-tests. Frequency distributions or non-normal distributed parameters will be compared between groups using non-parametric tests (chi-square, Mann-Whitney or Kruskal Wallis). Statistical tests will be done at the 5% significance level and where appropriate corresponding 95% confidence intervals will be presented. Data will be handled and analysed using SPSS Statistics version 24.0.0.1 (IBM Corp., Armonk, NY) and R, version 3.4.3.

(Objective A) To study patterns of co-occurrence of NCDs in the general population.

We will quantify the number of events for each disease separately and visualized all observed combinations of NCD co-occurrence during follow-up using an intersection-diagram.

(Objective B) To calculate the overall lifetime risk of developing any NCD.

When individuals are followed for long time periods (e.g., from mid-life to disease or death), preclusion of disease-specific outcomes of interest by death from other causes or precluding events from competing events may overestimate absolute risks in standard Kaplan-Meier analyses. To overcome the issue of such competing risks, we will analyze the data taking into account the occurrence of competing events to compute remaining lifetime risks in left truncated data with age as a time scale. Lifetime risk estimates reflect the competing risk-adjusted cumulative incidences from that particular age onwards until the age of last observation.

We will calculate the combined cumulative incidences of these diseases from the age of 45 to the age of last observation. The combined cumulative incidence equals the remaining lifetime risk of developing any NCD from the age of 45 onwards. For these analyses, follow-up will start at study entry (with the age of 45 y as minimum) and will end at the first date of diagnosis of any of these NCDs, death due to other causes than NCDs, lost to follow-up, or the administrative study end date of 1-1-2012. This means that we will consider only the first occurring event of the six potential outcomes in this analysis.

(Objective C) To calculate disease-specific lifetime risks of the different manifestations of NCDs.

As a complementary analysis, we will also calculated the disease-specific cumulative incidence in which we will consider only the disease of interest as outcome, while censoring for any of the five other diseases. Thus, participants will remain at risk of the six diseases irrespective of the occurrence of a first event. This means that participants with heart disease during follow-up in the stroke analysis, will remain at risk for stroke.

(Objective D) To study associations of three common shared risk factors, namely smoking, hypertension and overweight, with lifetime risk, age at onset, and life expectancy with and without NCDs.

We will stratify on risk factors at baseline (all three risk factors present vs. no risk factors present) to study the association of these risk factors with lifetime risk of any NCD. To assses the relation with the age at onset of any NCD, we will compare the two cumulative incidence curves of these groups (all three versus no risk factors at baseline), and calculate the difference between the two. This equals the time in years that the 'no risk factor' group requires to reach the same level of cumulative incidence as the 'all three risk factor' group.

To calculate the life expectancy with and without NCDs, we used multistate lifetables. This demographic tool combines all the life experiences of participants in three different health states: free of NCD, NCD, and death. Transitions between these states could be from free of NCD to NCD (incident NCD), NCD to death (mortality among individuals with NCD) and free of NCD to death (non-NCD mortality among individuals without NCD). We will consider only the first event into a state and backflows will not be allowed. We will adjust for age, sex, birth year, marital status, and educational level. First, we will calculate sex- and age-specific incidence rates. Second, we will obtain the prevalence of individuals with all three leading risk factors and without all these risk factors by 10-year age groups, separately for individuals with and without NCD. Subsequently, we will compute sex-specific hazard ratios (HRs) using Poisson regression with “Gompertz” distribution, while adjusting for the aforementioned covariates. The final transition rates for the two groups will be calculated using the abovementioned prevalence rates, overall transition rates, and adjusted HRs for NCD, and mortality. The multistate lifetables will start at age 45 and close at age 100. To calculate confidence intervals, we will use Monte Carlo simulation (parametric bootstrapping) with 10,000

iterations. Bootstrap calculations to compute confidence intervals for the multistate life expectancy calculations will be done with @RISK software (Palisade Corp., Middlesex, United Kingdom).
