# Supplementary material for: Lifetime risk and multimorbidity of non-communicable diseases and disease-free life expectancy in the general population: A population-based cohort study
Source: PLoS Med. 2019 Feb 4;16(2):e1002741. doi: 10.1371/journal.pmed.1002741 (PMC6361416; doi:10.1371/journal.pmed.1002741)
Supplement: S1 Methods — (DOCX) [file pmed.1002741.s003.docx]

**Lifetime risk and multimorbidity of non-communicable diseases and disease-free life expectancy in the general population: A population-based cohort study**

Silvan Licher^1^, Alis Heshmatollah^1,2^, Kimberly D. van der Willik^1,3^, Bruno H. Ch. Stricker^1^, Rikje Ruiter^1^, Emmely W. de Roos^1,4^, Lies Lahousse^1,5^, Peter J. Koudstaal^2^, Albert Hofman^6^, Lana Fani^1^, Guy G. O. Brusselle^1,4,7^, Daniel Bos^1,6,8^, Banafsheh Arshi^1^, Maryam Kavousi^1^, Maarten J. G. Leening^1,6,9^, M. Kamran Ikram^1,2‡^, M. Arfan Ikram^1‡^*

**1** Department of Epidemiology, Erasmus MC–University Medical Center Rotterdam, Rotterdam, the Netherlands,

**2** Department of Neurology, Erasmus MC–University Medical Center Rotterdam, Rotterdam, the Netherlands,

**3** Department of Psychosocial Research and Epidemiology, Netherlands Cancer Institute, Amsterdam, the Netherlands,

**4** Department of Respiratory Medicine, Erasmus MC–University Medical Center Rotterdam, Rotterdam, the Netherlands,

**5** Department of Bioanalysis, Faculty of Pharmaceutical Sciences, Ghent University, Ghent, Belgium,

**6** Department of Epidemiology, Harvard T.H. Chan School of Public Health, Boston, Massachusetts, United States of America,

**7** Department of Respiratory Medicine, Ghent University Hospital, Ghent, Belgium,

**8** Department of Radiology and Nuclear Medicine, Erasmus MC–University Medical Center Rotterdam, Rotterdam, the Netherlands,

**9** Department of Cardiology, Erasmus MC–University Medical Center Rotterdam, Rotterdam, the Netherlands

* m.a.ikram@erasmusmc.nl

‡ These authors are joint senior authors on this work.

**Abbreviation:** NCD, non-communicable disease

**Part A.** Ascertainment Methods of Non-communicable Diseases.……………..…...…….3
**Part B.** Life Expectancy Calculations ……………...….....………………………………..5**Supplement Part A.** Ascertainment Methods of Non-communicable Diseases

**Stroke**

Stroke was defined according to the World Health Organization (WHO) criteria as a syndrome of rapidly developing clinical signs of focal (or global) disturbance of cerebral function.[1] Symptoms had to last 24 hours or longer or leading to death, with no apparent cause other than vascular origin. History of stroke was assessed at baseline and subsequent home interviews, and verified by reviewing medical records.[2] During follow-up, individuals were continuously monitored for incident stroke through automated linkage of the study database with files from general practitioners.

**Heart Disease**

Heart disease was defined as a composite of coronary heart disease and heart failure. Definitions and procedures on the adjudication of cardiac outcomes have been described in detail previously.[3,4] In brief, coronary heart disease was defined myocardial infarction, and surgical or percutaneous coronary revascularization procedure (as a proxy for unstable or incapacitating angina).[4] Myocardial infarction was defined according to the triad of symptoms, indicative ECG changes, and cardiac biomarkers.[4] In accordance with the guidelines of the European Society of Cardiology, heart failure was defined as the combination of typical symptoms and signs, confirmed by objective evidence of cardiac dysfunction or a positive response to initiated treatment.[3,4]

**Diabetes**

Diabetes was defined as a fasting blood glucose concentration of 7.0 mmol/L or higher, a non-fasting blood glucose concentration of 11.1 mmol/L or higher (when fasting samples were unavailable), or the use of blood glucose-lowering drugs. This definition is in accordance with the WHO guidelines.[5,6] Diabetes were ascertained by use of general practitioners’ records (including laboratory glucose measurements), hospital discharge letters, and serum glucose measurements from Rotterdam Study visits. Information about the use of blood glucose-lowering drugs was obtained from both structured home interviews and pharmacy dispensing records.

**Chronic Respiratory Disease**

Chronic respiratory disease was defined as chronic obstructive pulmonary disease (COPD) or asthma. COPD was defined as either an obstructive spirometry without an asthma diagnosis, or a validated clinical diagnosis of COPD.[7,8] Asthma was defined as a validated clinical diagnosis of asthma conform the Global Initiative for Asthma guidelines.[9] The validation of asthma cases is described in detail previously.[10] Since 2002, spirometry was embedded within the core protocol of the Rotterdam Study visits. A Master Screen^®^ PFT Pro was used according to the American Thoracic Society /European Respiratory Society guidelines.[7] Reversibility was not tested. Airway obstruction was defined as a forced expiratory volume in one second / forced vital capacity ratio <0.7.[11]

**Cancer**

Cancer was classified according to the International Classification of Diseases, tenth edition. Only pathology confirmed cancer diagnoses were used for the present analysis. Non-melanoma skin cancers were not included in the definition of cancer for the analysis. Date of diagnosis was based on the date of biopsy (solid tumors), or laboratory assessment (hematologic tumors).

**Neurodegenerative Disease**

Neurodegenerative disease was defined as a composite of dementia and parkinsonism. Individuals were screened for dementia at baseline and subsequent center visits with the Mini-Mental State Examination and the Geriatric Mental Schedule organic level.[12] Those with a Mini-Mental State Examination score <26 or Geriatric Mental Schedule score >0 underwent further investigation and informant interview, including the Cambridge Examination for Mental Disorders of the Elderly. All individuals also underwent routine cognitive assessment. In addition, the entire cohort was continuously under surveillance for dementia through linkage of the study database with medical records from general practitioners and the regional institute for outpatient mental health care. A consensus panel led by a consultant neurologist established the final diagnosis according to standard criteria for dementia (DSM-III-R), Alzheimer's disease (NINCDS–ADRDA) and vascular dementia (NINDS-AIREN). Individuals were screened for parkinsonism and Parkinson’s disease at baseline using a structured interview and neurological screening examination.[13] All individuals who used antiparkinsonian drugs (Anatomical Therapeutic Chemical classification index N04), reported that they had Parkinson’s disease, or had at least one possible cardinal sign of parkinsonism at the neurological examination were invited for further evaluation. This consisted of a structured clinical work-up including the motor examination of the Unified Parkinson's Disease Rating Scale (UPDRS),[14] a neurologic examination by a research physician, standardized history taking and obtaining additional information from medical records. We used diagnostic criteria agreed upon in EUROPARKINSON (European Community Concerted Action on the Epidemiology of Parkinson's Disease).[15] During follow-up, we used four overlapping modalities to detect potential cases of parkinsonism: in-person screening, self-reporting of Parkinson’s disease during in-person interviews, antiparkinsonian medication use, and alerts from continuous monitoring of clinical records.

**Supplement Part B.** Life Expectancy Analysis

## **Figure 2.** Risk of common neurological diseases for 45-years-old men and women.

**Life Expectancy Analysis**

First, we calculated sex- and age-specific incidence rates. Second, we obtained the prevalence of individuals with all three leading risk factors and without all these risk factors by 10-year age groups, separately for individuals with and without NCD. Subsequently, we computed sex-specific hazard ratios (HRs) using Poisson regression with “Gompertz” distribution. We adjusted for age, sex, birth year, marital status, and education level. The final transition rates for the two groups were calculated using the abovementioned prevalence rates, overall transition rates, and adjusted HRs for NCD, and mortality. The multistate lifetables started at age 45 and closed at age 100. To calculate confidence intervals, we used Monte Carlo simulation (parametric bootstrapping) with 10,000 iterations. Bootstrap calculations to compute confidence intervals for the multistate life expectancy calculations were done with @RISK software (Palisade Corp., Middlesex, United Kingdom).

**References**

1. Hatano S. Experience from a multicentre stroke register: a preliminary report. Bull World Health Organ. 1976;54(5):541-53.

2. Wieberdink RG, Ikram MA, Hofman A, Koudstaal PJ, Breteler MM. Trends in stroke incidence rates and stroke risk factors in Rotterdam, the Netherlands from 1990 to 2008. Eur J Epidemiol. 2012;27(4):287-95.

3. Bleumink GS, Knetsch AM, Sturkenboom MC, Straus SM, Hofman A, Deckers JW, et al. Quantifying the heart failure epidemic: prevalence, incidence rate, lifetime risk and prognosis of heart failure The Rotterdam Study. Eur Heart J. 2004;25(18):1614-9.

4. Leening MJ, Kavousi M, Heeringa J, van Rooij FJ, Verkroost-van Heemst J, Deckers JW, et al. Methods of data collection and definitions of cardiac outcomes in the Rotterdam Study. Eur J Epidemiol. 2012;27(3):173-85.

5. World Health Organization. Definition and diagnosis of diabetes mellitus and intermediate hyperglycemia: report of a WHO/IDF consultation. Geneva: World Health Organization; 2006. p. 1-50.

6. Stolk RP, Pols HA, Lamberts SW, de Jong PT, Hofman A, Grobbee DE. Diabetes mellitus, impaired glucose tolerance, and hyperinsulinemia in an elderly population. The Rotterdam Study. Am J Epidemiol. 1997;145(1):24-32.

7. Ikram MA, Brusselle GGO, Murad SD, van Duijn CM, Franco OH, Goedegebure A, et al. The Rotterdam Study: 2018 update on objectives, design and main results. Eur J Epidemiol. 2017;32(9):807-50.

8. Vogelmeier CF, Criner GJ, Martinez FJ, Anzueto A, Barnes PJ, Bourbeau J, et al. Global Strategy for the Diagnosis, Management, and Prevention of Chronic Obstructive Lung Disease 2017 Report. GOLD Executive Summary. Am J Respir Crit Care Med. 2017;195(5):557-82.

9. Bateman ED, Hurd SS, Barnes PJ, Bousquet J, Drazen JM, FitzGerald JM, et al. Global strategy for asthma management and prevention: GINA executive summary. Eur Respir J. 2008;31(1):143-78.

10. de Roos EW, Lahousse L, Verhamme KMC, Braunstahl GJ, Ikram MA, In 't Veen J, et al. Asthma and its comorbidities in middle-aged and older adults; the Rotterdam Study. Respir Med. 2018;139:6-12.

11. Terzikhan N, Verhamme KM, Hofman A, Stricker BH, Brusselle GG, Lahousse L. Prevalence and incidence of COPD in smokers and non-smokers: the Rotterdam Study. Eur J Epidemiol. 2016;31(8):785-92.

12. de Bruijn RF, Bos MJ, Portegies ML, Hofman A, Franco OH, Koudstaal PJ, et al. The potential for prevention of dementia across two decades: the prospective, population-based Rotterdam Study. BMC Med. 2015;13:132.

13. Darweesh SK, Koudstaal PJ, Stricker BH, Hofman A, Ikram MA. Trends in the Incidence of Parkinson Disease in the General Population: The Rotterdam Study. Am J Epidemiol. 2016;183(11):1018-26.

14. Fahn S ER, Members of the UPDRS Development Committee. The Unified Parkinson's Disease Rating Scale. Florham Park, NJ: Macmillan Healthcare Information; 1987.

15. de Rijk MC, Tzourio C, Breteler MM, Dartigues JF, Amaducci L, Lopez-Pousa S, et al. Prevalence of parkinsonism and Parkinson's disease in Europe: the EUROPARKINSON Collaborative Study. European Community Concerted Action on the Epidemiology of Parkinson's disease. J Neurol Neurosurg Psychiatry. 1997;62(1):10-5.
