# Supplementary material for: Lifetime risk and multimorbidity of non-communicable diseases and disease-free life expectancy in the general population: A population-based cohort study
Source: PLoS Med. 2019 Feb 4;16(2):e1002741. doi: 10.1371/journal.pmed.1002741 (PMC6361416; doi:10.1371/journal.pmed.1002741)
Supplement: S1 Results — (DOCX) [file pmed.1002741.s004.docx]

**Lifetime risk and multimorbidity of non-communicable diseases and disease-free life expectancy in the general population: A population-based cohort study**

Silvan Licher^1^, Alis Heshmatollah^1,2^, Kimberly D. van der Willik^1,3^, Bruno H. Ch. Stricker^1^, Rikje Ruiter^1^, Emmely W. de Roos^1,4^, Lies Lahousse^1,5^, Peter J. Koudstaal^2^, Albert Hofman^6^, Lana Fani^1^, Guy G. O. Brusselle^1,4,7^, Daniel Bos^1,6,8^, Banafsheh Arshi^1^, Maryam Kavousi^1^, Maarten J. G. Leening^1,6,9^, M. Kamran Ikram^1,2‡^, M. Arfan Ikram^1‡^*

**1** Department of Epidemiology, Erasmus MC–University Medical Center Rotterdam, Rotterdam, the Netherlands,

**2** Department of Neurology, Erasmus MC–University Medical Center Rotterdam, Rotterdam, the Netherlands,

**3** Department of Psychosocial Research and Epidemiology, Netherlands Cancer Institute, Amsterdam, the Netherlands,

**4** Department of Respiratory Medicine, Erasmus MC–University Medical Center Rotterdam, Rotterdam, the Netherlands,

**5** Department of Bioanalysis, Faculty of Pharmaceutical Sciences, Ghent University, Ghent, Belgium,

**6** Department of Epidemiology, Harvard T.H. Chan School of Public Health, Boston, Massachusetts, United States of America,

**7** Department of Respiratory Medicine, Ghent University Hospital, Ghent, Belgium,

**8** Department of Radiology and Nuclear Medicine, Erasmus MC–University Medical Center Rotterdam, Rotterdam, the Netherlands,

**9** Department of Cardiology, Erasmus MC–University Medical Center Rotterdam, Rotterdam, the Netherlands

* m.a.ikram@erasmusmc.nl

‡ These authors are joint senior authors on this work.

**Abbreviation:** NCD, non-communicable disease

**Table A. Baseline characteristics of the study population, stratified by study wave**

|  | **RS-I wave**  (n=4,869) | **RS-II wave**  (n=1,620) | **RS-III wave**  (n=2,572) |
| --- | --- | --- | --- |
| Age, median (IQR) | 66.9 (61.2 – 73.9) | 61.3 (58.4 – 65.9) | 55.9 (51.5 – 59.7) |
| Sex, women | 3,048 (62.6%) | 922 (56.9%) | 1,488 (57.9%) |
| Marital status |  |  |  |
| Living with partner | 3156 (64,8%) | 1244 (76,8%) | 2027 (78,8%) |
| Living without partner | 1712 (35,2%) | 376 (23,2%) | 545 (21,2%) |
| Educational level |  |  |  |
| Primary | 1,073 (22.0%) | 123 (7.6%) | 240 (9.3%) |
| Lower | 2,016 (41.4%) | 717 (44.3%) | 880 (34.2%) |
| Further | 1,260 (25.9%) | 470 (29.0%) | 703 (27.3%) |
| Higher | 416 (8.5%) | 290 (17.9%) | 726 (28.2%) |
| Smoking status |  |  |  |
| Never | 1,751 (36.0%) | 512 (31.6%) | 809 (31.5%) |
| Former | 1,944 (39.9%) | 722 (44.6%) | 1,075 (41.8%) |
| Current | 1,121 (23.0%) | 380 (23.5%) | 679 (26.4%) |
| Systolic blood pressure (mm Hg) | 139 ±22 | 141 ±21 | 132 ±19 |
| Diastolic blood pressure (mm Hg) | 74 ±11 | 79 ±10 | 83 ±11 |
| Use of blood pressure lowering medication | 1,228 (25.2%) | 366 (22.6%) | 498 (19.4%) |
| Hypertension | 2,667 (54.8%) | 905 (55.9%) | 1,124 (43.7%) |
| Body-mass index (kg/m^2^) | 26.2 ±3.6 | 26.8 ±3.9 | 27.2 ±4.2 |
| Overweight | 2,880 (59.1%) | 1,081 (66.7%) | 1,718 (66.8%) |

Data presented as frequency (percent) for categorical values and mean ± SD for continuous variables, unless indicated otherwise. Abbreviations: RS: Rotterdam Study, SD: standard deviation, IQR: interquartile range.

**Table B. Lifetime Risks for any NCD, Stratified by Sex and Study Wave**

|  | **RS – I, % [95% CI]** | **RS – II, % [95% CI]** | **RS – III, % [95% CI]** | ***P*-for-difference** |
| --- | --- | --- | --- | --- |
| Men | 92.0 [90.7 – 93.7] | 95.6 [92.6 – 98.7] | 95.6 [90.4 – 100.0] | <0.001 |
| Women | 91.9 [90.7 – 93.1] | 94.1 [91.0 – 97.2] | 82.5 [71.0 – 94.1] | <0.001 |

In this analyses, follow-up ended at time of first occurrence of an noncommunicable disease. For instance, for individuals who first experienced heart disease and subsequently developed neurodegenerative disease, only heart disease is considered here.

**Table C. Remaining Lifetime Risk at age 45 for any NCD, Stratified by the Presence of each Risk Factor Separately and their Possible Combinations.**

|  | **Lifetime Risk [95%]** |
| --- | --- |
| Current smoking | 94.3 [92.7 – 95.8] |
| No current smoking | 92.9 [92.1 – 93.7] |
|  |  |
| Hypertension | 94.2 [93.4 – 95.1] |
| No hypertension | 92.5 [91.3 – 93.8] |
|  |  |
|  |  |
| Overweight | 94.9 [94.1 – 95.6] |
| Not overweight | 90.5 [88.9 – 92.1] |
|  |  |
|  |  |
| Current smoking and hypertension | 95.4 [93.9 – 96.9] |
| No current smoking or hypertension | 93.0 [92.2 – 93.8] |
|  |  |
|  |  |
| Current smoking and overweight | 97.1 [96.0 – 98.1] |
| No current smoking or overweight | 92.5 [91.6 – 93.4] |
|  |  |
|  |  |
| Overweight and hypertension | 95.2 [94.3 – 96.0] |
| No overweight or hypertension | 92.1 [91.0 – 93.1] |
|  |  |
|  |  |
| Current smoking, hypertension and overweight | 96.8 [95.3 – 98.2] |
| No current smoking, hypertension or overweight | 93.0 [92.2 – 93.8] |

**Table D. Lifetime Risks for Each NCD Separately, Stratified by Risk Factor Burden**

| **Noncommunicable Disease** | **None of the Three Risk Factors, % [95% CI]** | **All Three Risk**  **Factors, % [95% CI]** | ***P* -for-difference** |
| --- | --- | --- | --- |
| Stroke | 16.4 [12.5 – 20.3] | 25.8 [20.5 – 31.0] | 0.002 |
| Heart Disease | 36.6 [30.8 – 42.4] | 56.4 [49.6 – 63.3] | <0.001 |
| Diabetes | 14.6 [8.5 – 20.7] | 36.3 [28.4 – 44.1] | <0.001 |
| Chronic Respiratory Disease | 19.2 [15.6 – 23.0] | 39.9 [32.0 – 47.8] | <0.001 |
| Cancer | 40.1 [35.1 – 45.1] | 44.5 [38.0 – 51.1] | 0.143 |
| Neurodegenerative Disease | 40.0 [34.8 – 45.3] | 19.9 [14.9 – 24.8] | <0.001 |

In these analyses, individuals remained at risk of the specific NCD under study, irrespective of the occurrence of other NCDs, e.g. individuals with an incident stroke or heart disease were still at risk of diabetes.

**Table E. Remaining Life Expectancy at Age 45 with and without NCDs, Stratified by the Presence of each Risk Factor Separately and their Possible Combinations.**

|  | **Total life expectancy in years [95% CI)** | **Years lived without NCD [95% CI)** | **Years lived**  **with NCD [95% CI)** |
| --- | --- | --- | --- |
| Current smoking | 30.4 [30.1 – 30.7] | 20.0 [19.7 – 20.3] | 10.4 [10.1 – 10.8] |
| No current smoking | 34.8 [34.6 – 34.9] | 24.5 [24.3 – 24.7] | 10.2 [10.0 – 10.4] |
|  |  |  |  |
| Hypertension | 32.2 [32.0 – 32.4] | 21.3 [21.0 – 21.5] | 10.9 [10.7 – 11.1] |
| No hypertension | 34.4 [34.1 – 34.7] | 24.0 [23.7 – 24.3] | 10.5 [10.2 – 10.8] |
|  |  |  |  |
|  |  |  |  |
| Overweight | 31,2 [31.0 – 31.4] | 21.3 [21.1 – 21.5] | 9.9 [9.7 – 10.1] |
| Not overweight | 31.1 [30.8 – 31.4] | 22.1 [21.8 – 22.4] | 9.0 [8.7 – 9.3] |
|  |  |  |  |
|  |  |  |  |
| Current smoking and hypertension | 28.7 [28.3 – 29.1] | 18.2 [17.8 – 18.7] | 10.5 [9.9 – 11.0] |
| No current smoking or hypertension | 33.3 [33.2 – 33.4] | 22.6 [22.5 – 22.8] | 10.7 [10.6 – 10.8] |
|  |  |  |  |
|  |  |  |  |
| Current smoking and overweight | 29.2 [28.8 – 29.7] | 18.4 [18.0 – 18.9] | 10.8 [10.3 – 11.3] |
| No current smoking or overweight | 33.0 [32.9 – 33.1] | 22.9 [22.7 – 23.1] | 10.1 [10.0 – 10.3] |
|  |  |  |  |
|  |  |  |  |
| Overweight and hypertension | 31.3 [31.1 – 31.5] | 20.5 [20.2 – 20.8] | 10.8 [10.5 – 11.1] |
| No overweight or hypertension | 32.6 [32.4 – 32.8] | 22.5 [22.3 – 22.7] | 10.1 [9.9 – 10.4] |
|  |  |  |  |
|  |  |  |  |
| Current smoking, hypertension and overweight | 32.4 [32.0 – 32.9] | 22.2 [21.6 – 22.7] | 10.3 [9.7 – 10.8] |
| No current smoking, hypertension or overweight | 38.4 [37.9 – 39.0] | 30.1 [29.5 – 30.7] | 8.3 [7.8 – 8.8] |

Abbreviations: NCD: noncommunicable disease

**
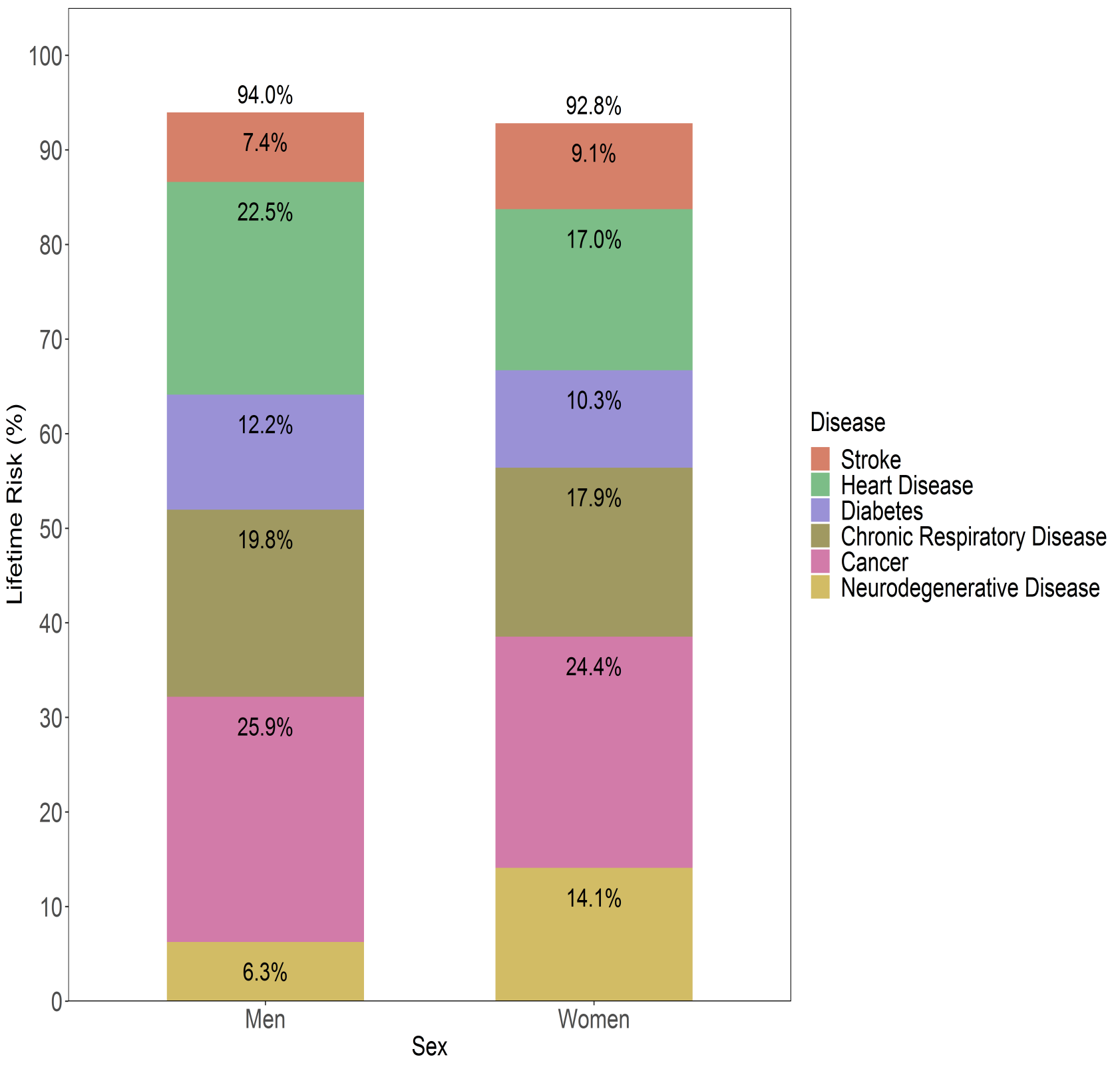
**

**Fig A. Lifetime Risk of NCDs for 45-years-old Men and Women**

In this analysis, follow-up ended at time of first occurrence of an noncommunicable disease. For instance, for individuals who first experienced heart disease and subsequently developed neurodegenerative disease, only heart disease is considered here.


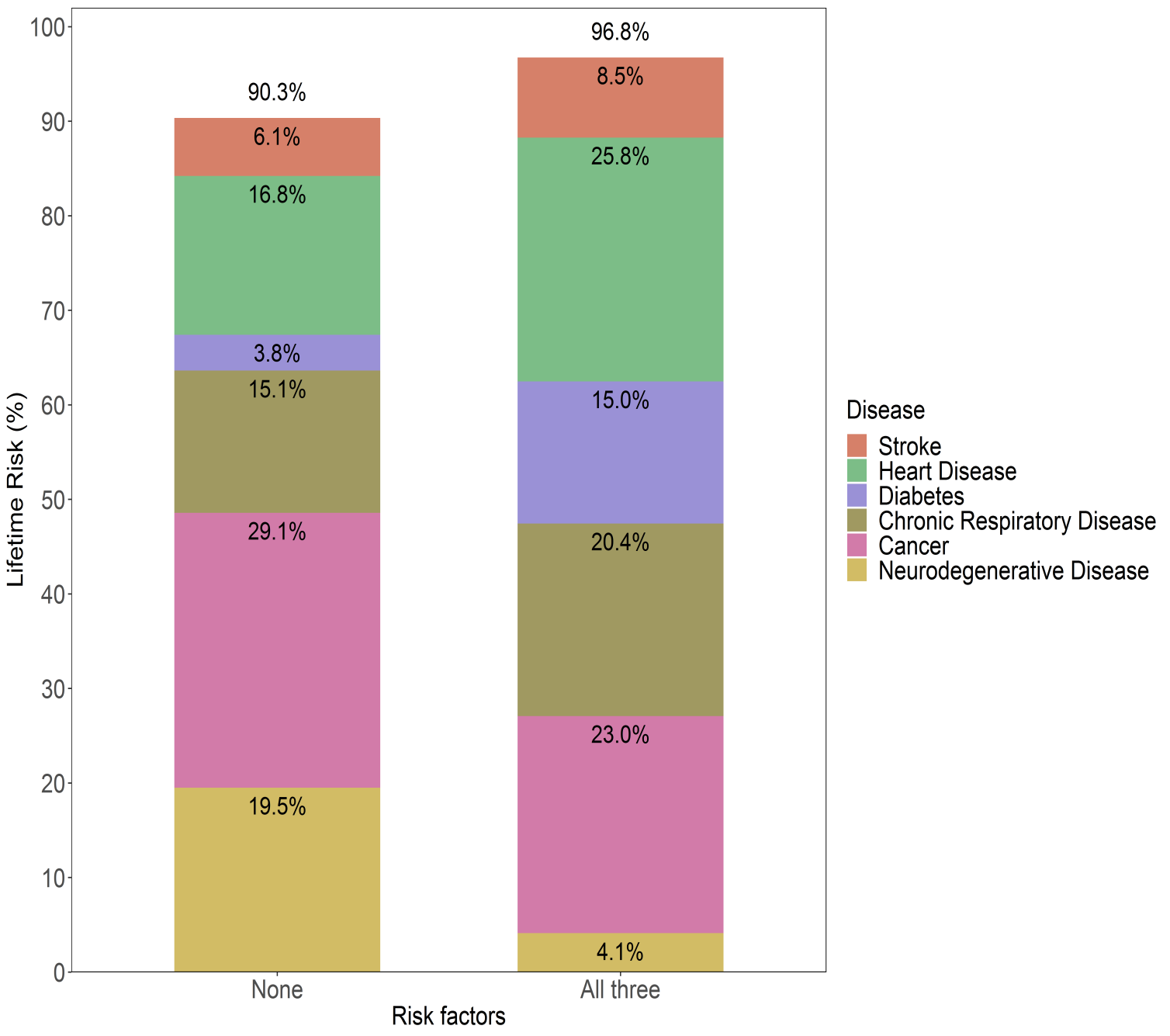
 **Fig B. Lifetime Risk of NCDs Among Those with All Optimal or Most Adverse Levels of Studied Risk Factors**

In this analysis, follow-up ended at time of first occurrence of a noncommunicable disease. For instance, for individuals who first experienced heart disease and subsequently developed neurodegenerative disease, only heart disease is considered here.


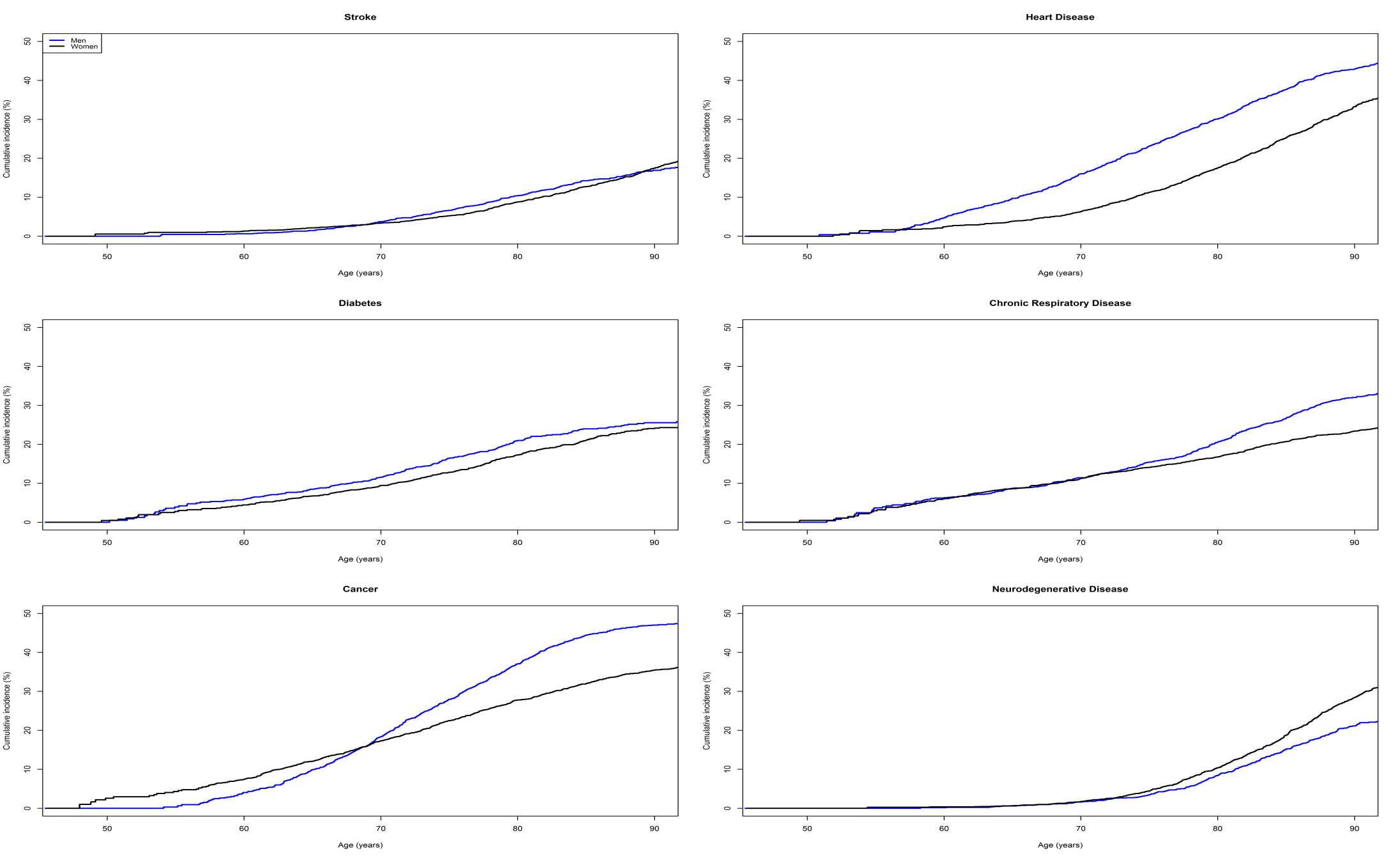
**Fig C.** **Cumulative Incidences for Each NCD Separately, Stratified by Sex**

In these analyses, individuals remained at risk of the specific NCD under study, irrespective of the occurrence of other NCDs, e.g. individuals with an incident stroke or heart disease were still at risk of diabetes.

**
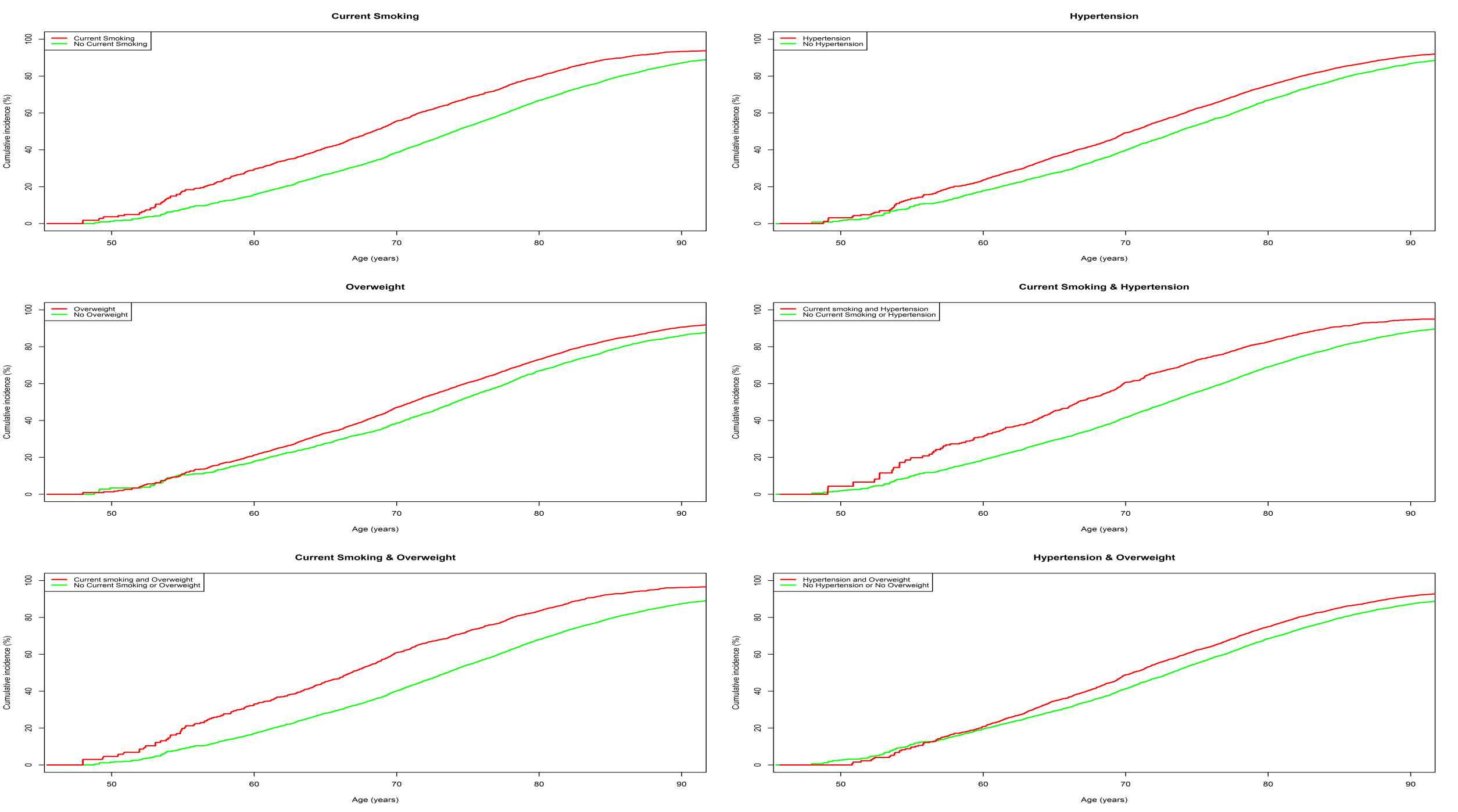
Fig D.** **Cumulative Incidence for any NCD, Stratified by Individual and Combinations of Risk Factor Strata**

In these analyses, follow-up ended at time of first occurrence of an NCD. For instance, for individuals who first experienced heart disease and subsequently developed neurodegenerative disease, only heart disease is considered here.


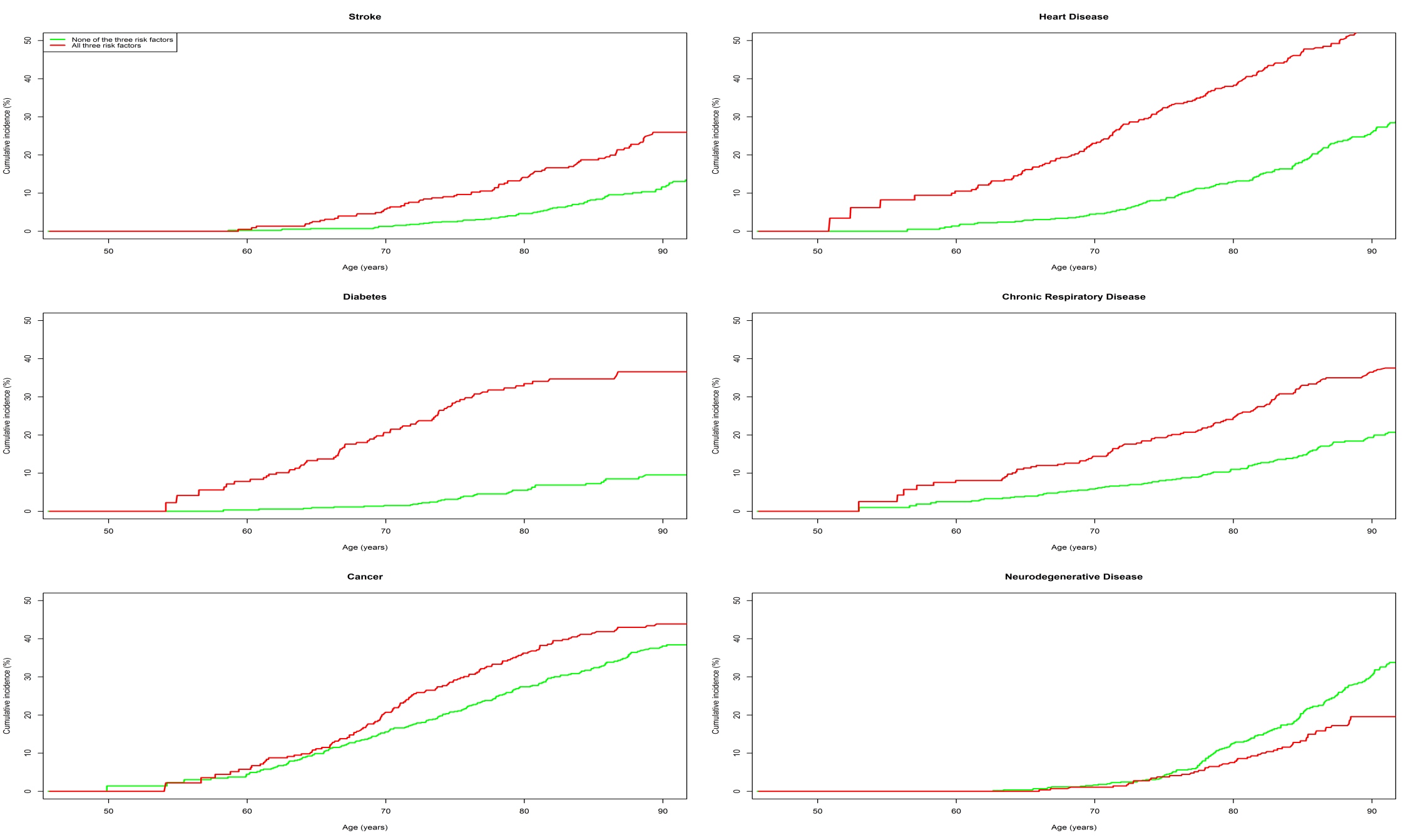
**Fig E.** **Cumulative Incidences for Each NCD Separately, Stratified by Risk Factor Strata**

In these analyses, individuals remained at risk of the specific NCD under study, irrespective of the occurrence of other NCDs, e.g. individuals with an incident stroke or heart disease were still at risk of diabetes.

**Fig F. Differences in Life Expectancy Depicted in Years for Individuals With at Least One Risk Factor, at Least Two Risk Factors and All Risk Factors Compared to the Life Expectancy of Individuals Without All three Risk Factors**

The reference group [life expectancy for persons without all risk factors) vary marginally due differences in model fit.

**Fig G. Differences in Life Expectancy Depicted in Percentages for Individuals With at Least One Risk Factor, at Least Two Risk Factors and All Risk Factors Compared to the Life Expectancy of Individuals Without All three Risk Factors**

The reference group [life expectancy for persons without all risk factors) vary marginally due differences in model fit.
